# Optimized Combination of HDACI and TKI Efficiently Inhibits Metabolic Activity in Renal Cell Carcinoma and Overcomes Sunitinib Resistance

**DOI:** 10.3390/cancers12113172

**Published:** 2020-10-28

**Authors:** Magdalena Rausch, Andrea Weiss, Marloes Zoetemelk, Sander R. Piersma, Connie R. Jimenez, Judy R. van Beijnum, Patrycja Nowak-Sliwinska

**Affiliations:** 1 Molecular Pharmacology Group, School of Pharmaceutical Sciences, University of Geneva, 1211 Geneva, Switzerland; Magdalena.Rausch@unige.ch (M.R.); andrea.weiss7@gmail.com (A.W.); Marloes.Zoetemelk@unige.ch (M.Z.); 2 Institute of Pharmaceutical Sciences of Western Switzerland, University of Geneva, 1211 Geneva, Switzerland; 3 Translational Research Center in Oncohaematology, 1211 Geneva, Switzerland; 4 Department of Medical Oncology, Amsterdam UMC, Vrije Universiteit Amsterdam, Medical Oncology, Cancer Center Amsterdam, De Boelelaan, 1117 Amsterdam, The Netherlands; s.piersma@vumc.nl (S.R.P.); c.jimenez@vumc.nl (C.R.J.); 5 OncoProteomics Laboratory, Cancer Center Amsterdam, Amsterdam UMC, Vrije Universiteit Amsterdam, 1117 Amsterdam, The Netherlands; 6 Angiogenesis Laboratory, Department of Medical Oncology, Cancer Center Amsterdam, Amsterdam UMC-Location VUmc, VU University Amsterdam, 1117 Amsterdam, The Netherlands; j.vanbeijnum@amsterdamumc.nl

**Keywords:** drug–drug interaction, HDAC inhibitor, multi-drug combination, renal cell carcinoma, therapeutically guided multidrug optimization (TGMO), tyrosine kinase inhibitor

## Abstract

**Simple Summary:**

To ameliorate the situation for kidney cancer patients and to broaden the application of available drugs, we initiated this research to enhance the anti-cancer activity through combination treatment. There is an unmet need for innovative treatment strategies and optimized drug combinations haven proven to be an adequate solution. We identified a four-drug combination of two histone deacetylate and two tyrosine kinase inhibitors that is effective in sunitinib-naïve and -resistant human renal cell carcinoma cells. Through our research, we demonstrated the superior anti-cancer activity of an optimized drug combination in comparison to single drugs, while maintaining a good safety/selectivity profile. We anticipate that the development and use of well-established drug combinations will be enforced offering personalized and more diverse treatment options in clinical conditions.

**Abstract:**

Clear cell renal cell carcinoma (ccRCC) is characterized by high histone deacetylase (HDAC) activity triggering both cell motility and the development of metastasis. Therefore, there is an unmet need to establish innovative strategies to advance the use of HDAC inhibitors (HDACIs). We selected a set of tyrosine kinase inhibitors (TKIs) and HDACIs to test them in combination, using the validated therapeutically guided multidrug optimization (TGMO) technique based on experimental testing and in silico data modeling. We determined a synergistic low-dose three-drug combination decreasing the cell metabolic activity in metastatic ccRCC cells, Caki-1, by over 80%. This drug combination induced apoptosis and showed anti-angiogenic activity, both in original Caki-1 and in sunitinib-resistant Caki-1 cells. Through phosphoproteomic analysis, we revealed additional targets to improve the translation of this combination in 3-D (co-)culture systems. Cell–cell and cell–environment interactions increased, reverting the invasive and metastatic phenotype of Caki-1 cells. Our data suggest that our optimized low-dose drug combination is highly effective in complex in vitro settings and promotes the activity of HDACIs.

## 1. Introduction

The reformed landscape of histone modifications in cancer modifies the plasticity of the DNA, promotes infinite cell cycle progression, and allows the uncontrolled transcription of (onco-) genes [1]. Drugs targeting these aberrant epigenetic processes have emerged as an effective therapeutic strategy and therefore, histone deacetylase inhibitors (HDACIs) nowadays play an important role in combating cancer [2]. HDACIs inhibit the removal of acetyl residues from histones by binding to all or subtypes of histone deacetylases (HDACs), hence remodeling the landscape of histone modifications to a ‘natural’ state [1]. Nonetheless, only four are currently FDA approved, three for the treatment of T cell lymphoma [3,4,5] and one for the treatment of multiple myeloma [6], and only two phase III trials have been launched validating HDACIs as therapeutics for breast cancer [7], or prostate cancer [8]. The European Medicines Agency (EMA), on the contrary, approved exclusively one HDACI for the treatment of multiple myeloma and refused another HDACI, romidepsin, for the treatment of non-Hodgkin lymphoma [9].

It appears that there is a gap between pre-clinical research and clinical translation in the use of HDACIs for the treatment of cancers with a strong dependency on HDAC function. In renal cell carcinoma (RCC), HDACs determine cell invasion, motility, and metastatic behavior [10] accompanied by facilitating metastasis-associated epithelial–mesenchymal transition [11]. HDACs play another important role in the epigenetic silencing of the Von Hippel–Lindau gene (*vhl*), harboring the driver mutation to advance RCC by dysregulating hypoxia-inducible factors (HIFs) [12]. In addition, cell adhesion molecules and growth factor receptors, e.g., E-cadherin and platelet-derived growth factor receptor-β, are key factors in forming metastasis and their expression can be reduced through the administration of HDACIs [13]. Moreover, valproic acid, inhibiting HDAC2, a class-I HDAC located in the cell nucleus, proved to be a good candidate for the treatment of RCC by changing the cell proliferation rate via histone hyper-acetylation and subsequent regulation of cell cycle proteins that, in turn, promote the cell cycle progression [14]. Consequently, HDACs are considerable targets in RCC with the application of HDACIs by simultaneously provoking a reduction of proliferation and inducing apoptosis [15].

Modernized approaches for medical care seek to introduce HDACIs successfully to clinical use by combining them with (i) DNA repair-targeting agents, (ii) radiotherapy, (iii) topoisomerase inhibitors, (iv) chemotherapy, (v) proteasome inhibitors, (vi) tyrosine kinase inhibitors (TKIs), or (vii) immune checkpoint inhibitors [16]. As tyrosine kinases and coupled signaling pathways play an important role in promoting the activation of HDACs, and several TKIs have been approved for the treatment of RCC, the combination of HDACIs and TKIs might hold the greatest benefits. The HDACI panobinostat in combination with erlotinib (TKI) has been tested in a phase I clinical study for the treatment of advanced aero-digestive tract tumors, i.e., non-small lung cell cancer, head and neck cancer. So far, no further studies have been conducted to validate this combination, as the primary results were inconclusive and a bigger sample size was necessary [17]. It has further been shown that the cytotoxic efficacy of sorafenib (TKI) against RCC can be synergistically enhanced through combination with either belinostat (HDACI) or vorinostat (HDACI) [18]. This pronounced interaction of these agents with sorafenib has been used to initiate different clinical studies for varying cancer types (ClinicalTrials.gov Identifiers: NCT01159301, NCT00823290, NCT01005797, NCT01075113, NCT00635791), but until now, none of them have been approved for the treatment of RCC. In addition, panobinostat or vorinostat are under clinical investigation while combined with known and novel proteasome inhibitors [19,20]. We have recently shown that cell-specific screening for drug combinations including HDACIs for the treatment of RCC revealed a highly synergistic four-drug combination [21]. In particular, two HDACIs (tacedinaline, tubacin) were combined with two TKIs (dasatinib, erlotinib) and tested in vitro in three human RCC cell lines, outperforming the single use of TKI sunitinib (Sutent^®^), the clinically used first-line treatment for RCC. The combination of tacedinaline, tubacin, dasatinib, and erlotinib showed high efficacy in cells with aberrant but functional proliferation due to the presence of supernumerary centrosomes forming multipolar spindles. However, not all ccRCC lesions present this phenotype or a similar genetic landscape, i.e., *vhl* mutation, and might remain insensitive to this four-drug combination.

In our search for a cell-specific selective drug combination, we used our validated therapeutically guided multidrug optimization (TGMO) technique. This phenotypically driven method determines synergistic multidrug combinations [21]. It allows us to merge the experimental data points with computational modeling to select for drugs, which interact synergistically while excluding those drugs that interact antagonistically [21,22].

In this study, we applied the TGMO technique, followed by an integration of genomics and phosphoproteomic analysis, and we identified a four-drug combination of two HDACIs and two TKIs, namely panobinostat, vorinostat, axitinib, and pictilisib, optimized for Caki-1 cells. Panobinostat and vorinostat are pan-HDACIs, inhibiting the catalytic activity to remove acetyl residues from DNA-bound histones, while axitinib is a TKI binding to the vascular endothelial growth factor receptor (VEGFR), leading to the blockade of further cellular signal transduction. Through the addition of pictilisib (TKI), the anticancer activity of the ODC could be further enhanced to efficiently reduce the viability of Caki-1 cells being naïve or resistant to the treatment with sunitinib (TKI) cultured in 3-D homotypic, as well as heterotypic spheroids.

## 2. Results

### 2.1. TGMO-Based Screen and Multidrug Combination Optimization Process

**Figure 1:**
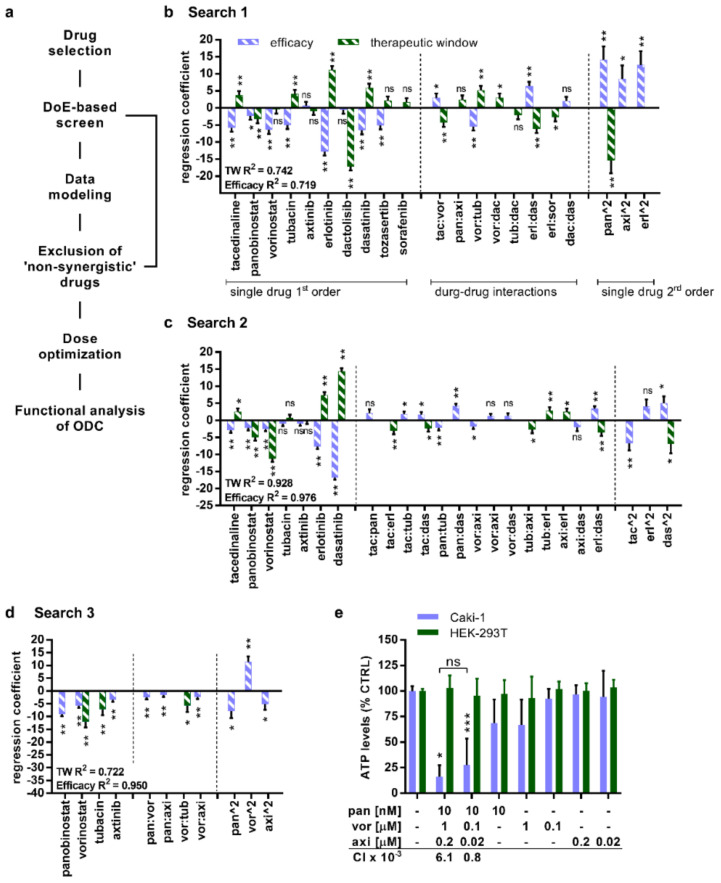
Therapeutically guided multidrug optimization (TGMO) selects synergistically interacting drugs in a multidrug combination active in Caki-1 cells. (**a**) Scheme of the TGMO method. Regression coefficients express activities or interactions of the drugs selected for the (**b**) Search 1 (155 multi-drug combinations) in the TGMO screen (*N* = 2). To distinguish simultaneously the efficacy (violet striped bars) and the therapeutic window (green bars), the TGMO screen was performed in Caki-1 cells and to obtain a therapeutic window in non-malignant human embryonic kidney (HEK-293T) cells. (**c**) Search 2, where 50 multidrug combinations were tested experimentally (*N* = 2). (**d**) Search 3, 25 multidrug combinations (*N* = 2). (**e**) The activity and selectivity of the optimized multidrug combination, measured as the level of ATP in comparison to the CTRL (0.03% dimethylsulfoxid (DMSO) in culture medium) was analyzed in Caki-1 and HEK-293T cells (*N* = 5). The level of ATP is a measure of cell viability. The combinatorial index (CI) was calculated for the multidrug combination at selected doses related to Figure S6, representing that both combinations are synergistic (CI < 1). Error bars represent the SD and significance of regression coefficients was determined with a one-way ANOVA and is represented with * *p* < 0.05, ** *p* < 0.01, and *** *p* < 0.001. R2 represents model accuracy in a coefficient of multiple determinations. ns, not significant.

The therapeutically guided multidrug optimization (TGMO) technique [21,22] (Figure 1a and Text S1) was established to identify selective multidrug combinations for the treatment of cancer. Guided through the cellular biology on phenotypic changes in response to the drug treatment, this technique connects an experimental design and in silico data modeling to select the optimal drugs in combination. The TGMO is based on orthogonally designed multidrug experiments performed in three consecutive search rounds to optimize a cell type-specific synergistic multidrug combination. Within the search, the drugs that do not add a synergistic effect to the overall combination are being excluded from further investigation. In addition, by subtracting the drug combination effect on cancer cells a therapeutic window (TW) is established and modeled in the TGMO-based screen and used as a parameter of selection.

To initiate the TGMO-based screen, we first selected a set of drugs including four HDACIs (tacedinaline, panobinostat, vorinostat, and tubacin), four TKIs (axitinib, erlotinib, dactolisib, dasatinib), and two serine-threonine kinase inhibitors (sorafenib, tozasertib) (Figure S1 and Table S1). Tacedinaline, panobinostat, and vorinostat block the catalytic activity of class I and class II HDAC, while tubacin exclusively inhibits HDAC6. The TKIs axitinib and erlotinib bind to extracellular growth factor receptors; dactolisib and dasatinib interfere with intracellular signaling proteins located at various levels of the mitogen-activated protein kinase (MAPK) pathway, whereas sorafenib inhibits at both levels. Tozasertib interacts with aurora kinases that regulate cell proliferation. Our selection was based on the following considerations: (i) the drugs have a known mechanism of action, (ii) synergistic interactions with other drugs have been reported, and (iii) (pre-)clinical studies have determined an existing anticancer activity in (cc)RCC. Further, these drugs were selected because they are in clinical evaluation or approved, with their pharmaco-kinetics/-dynamics being described, which allowed determination of the doses administered and measured in the blood plasma of the patients in the clinical settings. Another important criterion for the inclusion in this study was the established safety/toxicity profile.

We selected the human metastatic ccRCC cell line Caki-1 (Table S2) and evaluated the dose-response to each of the drugs (Figure S2). In parallel, we tested the single drugs at dose ranges adjusted to the clinically used doses (CUDs) in non-cancerous HEK-293T cells (Figure S2) and calculated the effective doses (EDs) reducing the cell viability by 20% (ED20) and ED10. These doses were used in the TGMO-based screen [21,22] (Figure 1a).

The data of these in vitro performed experiments facilitate the mathematical calculation of second-order linear regression models, leading to a subsequent drug-dose search (Supplementary Information S1). Regression coefficients representing “single-drug first order”, “two-drug interactions”, and “single-drug second-order” terms characterize linear regression models generated from data in each “Search” (Figure 1b–d and Figure S3). A coefficient of determination close to 1 describes a strong correlation between the experimentally gathered data points and the predicted data points, obtained through the modeling. Through this technique, sequentially from “Search 1” to “Search 3” (Figure 1b–d), we were able to diminish the number of drugs based on the calculated drug-related parameters.

As a result, our search revealed an optimized drug combination (ODC) synergistically active at two different dose levels. The doses are lower or relate to the clinically used doses (Table S3). This combination consisted of two HDACIs, namely panobinostat (pan) and vorinostat (vor), as well as one TKI axitinib (axi). Already within the initial optimization, panobinostat demonstrated strong efficacy with a negative regression coefficient, implying a strong dose-dependent activity as a mean of the single drug second-order term (Figure 1e). The final evaluation of the ODCs showed that both synergistic ODCs reduce the cellular viability (Figure 1e), measured as ATP levels compared to the cell treated with the negative control (0.03% DMSO in culture medium, CTRL; see Materials and Methods, Section 4.1.) in Caki-1 but not in HEK-293T cells (Figure 1e). Although panobinostat (applied at 10 nM) and vorinostat (applied at 1 µM) alone reduced the viability of Caki-1 cells, this effect was remarkably lower than this induced by the ODCs.

Moreover, we calculated the combination index (CI) using CompuSyn (ComboSyn, Inc., https://www.combosyn.com/) (Figure S4 and Table S4), which defines the synergy, additivity, or antagonism within a drug combination. This information is important to identify drug–drug interactions, which enable the reduction of the final drug doses used in combination and the probability of acquired drug resistance. Notably, both drug combinations were synergistic (CI < 1; Figure 1e). Even though the effect of the drug combination at higher doses appeared to be promising, the calculation of the CI revealed that drug–drug interactions of the lower dosed combination are strongly synergistic (6.08 × 10^−3^ vs. 7.61 × 10^−4^) (Figure 1e, Table S4). Therefore, we selected the combination of panobinostat, vorinostat, and axitinib applied at the doses of 10 nM, 0.1 µM, and 0.02 µM, respectively, for further evaluation.

### 2.2. Selective Activity of the ODC in Sunitinib-Naïve and Sunitinib Pre-Treated Caki-1 Cells Inducing Apoptosis

**Figure 2:**
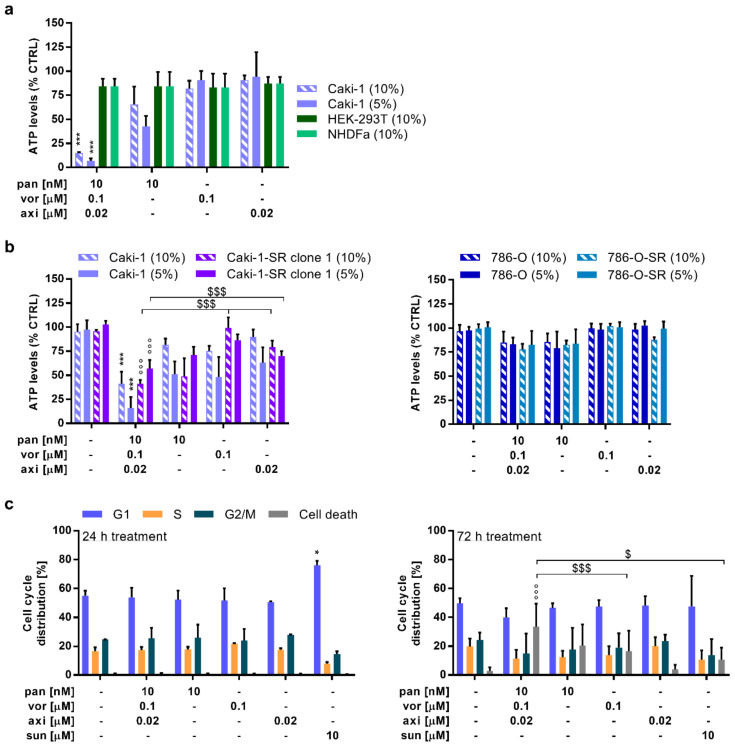
Assessment of the optimized drug combination (ODC) activity: selectivity profile, the efficacy to overcome sunitinib-acquired resistance, and the induction of cell death. (**a**) Cross-validation of the multidrug combination and the corresponding monotherapies in Caki-1 cells and non-malignant HEK-293T cells, and human fibroblasts (NHDFα). The treatment was applied on Caki-1 cells in medium supplemented with either 5% or 10% fetal bovine serum and on the non-malignant cell lines with 10% fetal bovine serum. (*N* = 3) (**b**) Validation of the anticancer activity in sunitinib-resistant (-SR) Caki-1 cells, as well as primary ccRCC cell line 786-O and 786-O-SR cells. (*N* = 3) (**c**) Flow cytometry analysis elucidating G1, S, G2/M phase, and cell death with DNA binding propidium iodide (PI) after treating Caki-1 cells for 24 (left graph) and 72 h (right graph). (*N* = 3) Error bars represent the SD and the significance of estimated regression coefficients was determined with a one-way ANOVA and is represented with * *p* < 0.05 and *** *p* < 0.001 versus the CTRL (0.03% DMSO in culture medium) and the monotherapies. °°° *p* < 0.001 represents significance calculated versus the CTRL only whereas ^$^ *p* < 0.05 and ^$$$^ *p* < 0.001 represent significance calculated vs. the monotherapies only.

In the next step, we validated the ODC activity in various cell lines, i.e., HEK-293T, NHDFα (Figure 2a and Table S5), 786-O cells, as well as in Caki-1 cells supplemented with 5 or 10% foetal bovine serum (FBS). The ODC was inactive in HEK-293T as well as NHDFα cells, as measured by ATP levels (% CTRL) (Figure 2a; Table S5). Moreover, the ODC outperformed non-optimized drug combinations and provided the most beneficial activity/synergy profile (Figure S6 and Table S4).

Sunitinib is a small molecule-based drug binding with high affinity to vascular endothelial growth factor receptor 2 and platelet-derived growth factor receptor β and in clinical use as first-line treatment for RCC. As a high number of patients develop treatment resistance to this drug [23], there is an utmost need to validate novel first- or second-line treatments in sunitinib-insensitive cells. To analyze the activity of the ODC in a more clinically relevant condition, we exposed Caki-1 and 786-O cells chronically treated with 1 µM sunitinib (-SR) (Figure 2b, left graph, Figure S5, and Table S2) to the ODC. Caki-1-SR cells were sensitive to the ODC, whereas 786-O (Table S2) and 786-O-SR cells (Figure 2b, right graph) were insensitive and potentially resistant to the ODC and its corresponding monotherapies.

Furthermore, we analyzed cell cycle distribution (G1, S, G2M, and cell death) following exposure to ODCs and monotherapies. We performed the analysis 24 and 72 h (Figure 2c) after treatment using flow cytometry for the DNA content. We did not detect any significant alteration in cell cycle distribution at 24 h post-treatment with ODC and monotherapies. However, in cells treated with 10 µM sunitinib, used here as a positive control, more cells were arrested in the G1 phase. Remarkably, 72 h post-treatment, the ODC, but not the monotherapies, specifically induced a significant level of cell death (57.2%). To investigate whether the inference of cell death is caused by proliferative dysregulation, cells were stained for the expression of Ki67 (Figure S7a), a marker strongly associated with tumor cell proliferation. This analysis revealed that compared to the CTRL, treatment with the ODC and also panobinostat significantly decreased the expression of Ki67 (76.35% and 72.48%; see Figure S7b).

### 2.3. The ODC Decreases Adherence and the Migratory Capacity of Metastatic ccRCC Cells

In the next step of the ODC efficacy validation, we used the homotypic 3-D spheroid cultures (3Dc) composed of Caki-1 cells. Validating the cytotoxic effect of the ODC in 3-Dc revealed that the ODC did not inhibit the cellular viability (Figure S8a). This suggests that the cytotoxic effect of panobinostat and axitinib is lower on 3-Dc than when performed in single-layer (2-D) cultures. Interestingly, the inhibitory effects of vorinostat on cellular viability were much stronger in 3-Dc than in 2-D single-layer cultures (Figure S8b). Further, the rigidity of the milieu had an impact on the cytotoxic effect of the combination. The addition of 25% basement membrane components (BM) increased the rigidity of the environment and thereby increased the cytotoxic efficacy of the ODC. In these conditions, however, the monotherapies of panobinostat and vorinostat were responsible for approximately 40% of ODC activity (Figure S8c).

**Figure 3:**
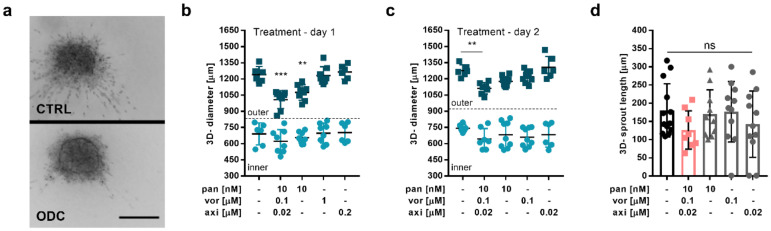
Inhibition of cell migration cultivated in 3-D spheroids. (**a**) Representative images of 3-D homotypic spheroids composed of Caki-1 cells cultured in a low attachment plate in 0.5 mg/mL collagen type I and treated with the CTRL (0.03% DMSO in culture medium, top image) and the multidrug combination (ODC, bottom image). Scale bar = 200 µm. The impact of the treatment conditions applied on (**b**) day 1 (24 h after seeding; *N* = 3) and (**c**) on day 2 (48 h after seeding; *N* = 3) on the spheroid growth was measured through the size of the diameter. (**d**) Treatment effect on the sprout length, (*N* = 3). Error bars represent the SD and significance was determined with a one-way ANOVA and is represented with ** *p* < 0.01 and *** *p* < 0.001. ns, not significant.

Simultaneously, these experiments unveiled that Caki-1 cells started to migrate when cultured in the presence of 0.5 mg/mL collagen type I, leading to the formation of sprouts (Figure 3a). The rigidity of this environment only facilitated the migration of the cells present at the surface of the spheroid but maintained a stable core of the spheroid. After either 24 (day 1) or 48 h (day 2) of spheroid formation, we administered the treatment for 72 h and validated characteristics related to migration. Representative pictures of spheroids treated with the CTRL or the ODC show that both spheroids have a dark inner core (inner) and a brighter outer margin (outer) (Figure 3a). The margin has been defined as the distance between the edge of the core spheroid (inner) and the migrating cells (outer). The enlargement of the margin overtime after treatment started after 24 or 48 h of spheroid formation and was measured until day 7 for all conditions tested (Figure S8e). The results were similar when administering the treatment after 24 (Figure 3b) or 48 h (Figure 3c) of spheroid formation; however, the activity mainly resulted from the presence of panobinostat reducing the size of the margin to almost the same extent (Figure 3b and Figure S8e).

Applying the ODC treatment on day 2, 48 h after spheroid formation, reduced the size of the outer area of the spheroid significantly (1275.8 vs. 1111.8 µm) compared to the CTRL. Besides, we observed that the ODC treatment reduced the single (180.7 vs. 126.5 µm) (Figure 3d) sprout length, though no significant changes were observed for the number of sprouts per spheroid (Figure S8d; left graph) or the number of branching points/mm^3^ (Figure S8d, right graph).

### 2.4. The Addition of MAPK Pathway-Inhibiting Drugs Enhances the Cytotoxic Activity of the ODC

**Figure 4:**
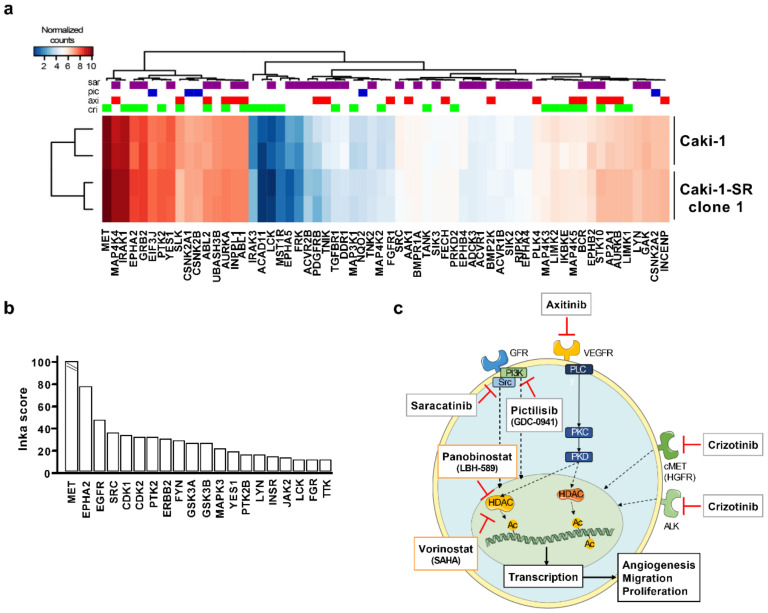
Importance of MET, MAPK, and Src signaling to enhance the cytotoxic activity of the multidrug combination and its translation. (**a**) Heat map of RNA expression in Caki-1 and sunitinib-resistant Caki-1 cells for the tyrosine kinase inhibitor targets as specified by ProteomicsDB. Drugs are color-coded as indicated. (**b**) Phosphoproteomic analysis of Caki-1 cells to determine potential drug targets. The INKA score on the *Y*-axis represents a measure of phosphotyrosine kinase activity. (**c**) Schematic drawing of the mechanism of action of the multidrug combination and MAPK-inhibiting agents selected through the RNA sequencing and phosphoproteomic analysis. Abbreviations: Ac-Acetyl; cMET–Tyrosine-protein kinase Met or (HGFR) hepatocyte growth factor receptor; HDAC–Histone deacetylase; PI3K–Phosphatidylinositol-3-kinase; PLC–Phospholipase Cγ; PKC–Phosphokinase C; PKD–Phosphokinase D; (VE)GFR–(Vascular endothelial) growth factor receptor; Src–Proto-oncogene tyrosine-protein kinase.

To identify additional drug targets to increase the activity of the ODC, we performed RNA sequencing (Figure 4a and Text S2) and phosphoproteomics (Figure 4b and Text S3) of Caki-1 cells. To cluster the transcripts in cellular/regulatory pathways, the 35 highest expressed transcripts (Table S6) were evaluated for their functional properties and drugability. In general, the RNA sequencing data showed that these transcripts, following analysis with the Gene Ontology Resource (GO; http://geneontology.org/) and Kyoto Encyclopedia of Genes and Genomes (KEGG; https://www.genome.jp/kegg/) databases, related to protein translation as well as adherence and motility (Table S6).

Tyrosine kinase drug targets, at specified concentrations, and established via a systematic screen of drug–protein interactions [24] were retrieved from the repository Proteomics DB (https://www.proteomicsdb.org/).

Drug targets of crizotinib, axitinib, saracatinib, and pictilisib were retrieved from the ProteomicsDB database. Subsequently, RNA expression data of these targets were extracted and presented in a heat map (Figure 4a). The drugs under study target a wide range of proteins expressed at different levels (Table S7). Aligning the RNA sequencing result with phosphoproteomics data revealed that the tyrosine kinases MET and Src show high transcript levels and are dominantly phosphorylated, together with the ephrin type-A receptor 2 (EPHA2) and EGFR (Figure 4b).

**Figure 5:**
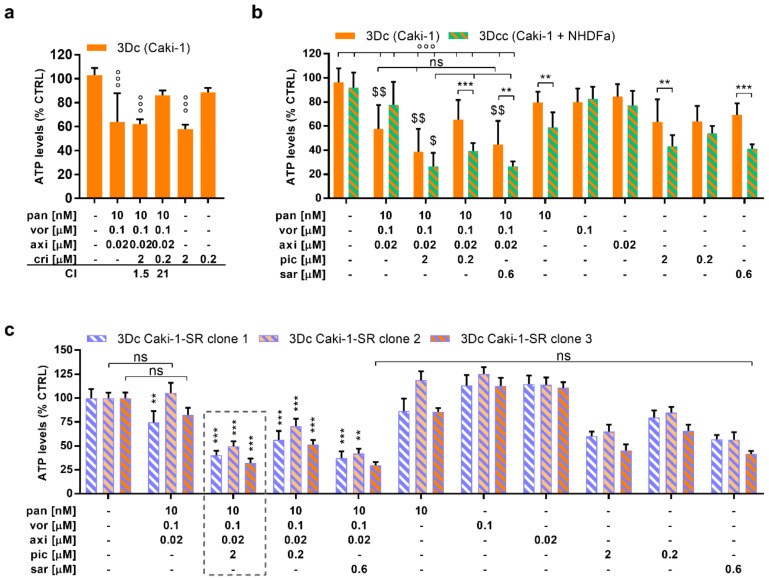
Activity of the multidrug combination after the addition of MAPK pathway-inhibiting drugs. (**a**) The activity of the multidrug combination of Caki-1 homotypic 3-D cultures (3-Dc) with and without the addition of crizotinib (cri) at two different doses. (*N* = 3) (**b**) Combinations of the original ODC and MAPK pathway-inhibiting small molecule drugs tested in Caki-1 3-D cultures and 3-D co-cultures (3-Dcc) of Caki-1 and NHDFα cells (*N* = 3). (**c**) Evaluation of the original ODC and other multidrug combination candidates supplemented with either pictilisib (pic) or saracatinib (sar) in three sunitinib-resistant Caki-1 clones (*N* = 3). Error bars represent the SD and significance was determined with (**a**,**b**) a one-way ANOVA or (**c**) a two-way ANOVA and is represented with ** *p* < 0.01 and *** *p* < 0.001 vs. the CTRL and the monotherapies. °°° *p* < 0.001 represents significance calculated vs. the CTRL only whereas ^$^ *p* < 0.05 and ^$$^ *p* < 0.01 represent significance calculated vs. the monotherapies only. ns, not significant.

During enhanced inter- and intracellular signaling, the mitogen-activated protein kinase (MAPK) cascade is highly upregulated in Caki-1 cells; therefore, we decided to add one TKI, with a distant target from axitinib, to the original combination. The selected drugs were saracatinib (sar), pictilisib (pic), or crizotinib (cri) at the doses of 0.6 and 2 µM. These doses reflect the clinically used doses. Crizotinib, a MET inhibitor, did enhance the cytotoxic activity but in an antagonistic fashion, being active as monotherapy (Figure 5a). Therefore, we validated the interplay of the original ODC by adding pictilisib (2 µM) or saracatinib (0.6 µM) in 3-D cultured (3-Dc) Caki-1 cells and 3-Dc co-cultured with NHDFα cells (3Dcc) (Figure 5b). The addition of pictilisib or saracatinib increased the cytotoxic activity of the ODC in the 3Dc to 61.4% and 55.2%, respectively. To discover the drug–drug interactions, we applied all variations of four-, three-, and two-drug combinations for 72 h on either 2-D (Figure S9) or 3-Dc (Figure S10) cultured Caki-1 cells.

The ODC was most active in 3-D co-cultured (3-Dcc) Caki-1 and NHDFα cells (Figure 5b), compared to three different sunitinib-resistant Caki-1 clones cultured in 3-Dc (Figure 5c), reducing the ATP levels by 48.1%. Regarding the overall anticancer activity measured in different assays, the ODC supplemented with pictilisib at a dose of 2 µM presented the strongest anticancer efficacy, significantly decreasing the ATP levels by ≥58.6%. The addition of 0.6 µM saracatinib to the ODC did diminish the cell viability; however, this activity results from the single activity of saracatinib. Of note, as erlotinib was already excluded through the original TGMO-based search, we did not include it at this step, despite the presence of EGFR in a top 20 INKA score (Figure 4b).

### 2.5. The ODC Possesses Anti-Angiogenic Activity, Reducing the Cell Viability of Endothelial Cells In Vitro and the Number of Vessel Branching Points In Vivo

The individual drugs composing the original ODC are known to also possess anti-angiogenic activity [25,26]. Indeed, the ODC revealed an anti-angiogenic efficacy, killing over 58% of human macrovascular endothelial (ECRF24) cells after 72 h of treatment (Figure S11a). The addition of either pictilisib or saracatinib did not improve the overall anti-angiogenic efficacy of the ODC (Figure S11b) in this model.

Using a two-dimensional migration assay (scratch assay), neither the ODC nor the monotherapies impaired the migratory behavior of Caki-1 or ECRF-24 cells. Sunitinib (10 µM), used here as a positive control, reduced effectively the migratory behavior of endothelial cells (65.8 vs. 35.6%) compared to the CTRL (Figure S11c).

Further, we evaluated the cell cycle distribution in ECRF24 cells (Figure S11d) upon treatment. The ODC induced cell death in 6.0% of the endothelial cells, which is 5.6-fold less than in Caki-1 cells.

Using the chorioallantoic membrane (CAM) of the chicken embryo (Figure S11e), a well-known model for studying angiogenesis, we observed a significant reduction in the number of vessel branching points/mm^3^ after the administration of the ODC compared to the CTRL (1049 vs. 1522) (Figure S11f,g).

## 3. Discussion

A promising strategy for future improvement of cancer therapies lies in the combination of various treatment options or multiple drugs that would help to turn non-responders into responders while simultaneously being safe. Combination therapies are currently being evaluated for cancer, infectious, and other complex diseases [27,28,29,30]. Combining drugs that target complementary pathways of the biological circuitry should be more effective in treating the highly adaptable cellular regulation [30,31]. The appearance of many new targeted pharmaceutical agents, and effective repurposing of drugs [32,33], offer tremendous potential for the creation of these drug cocktails. The major challenge in such a screen and identification of case-specific optimal drug cocktails is the massive number of possible drug-dose combinations. Today, there is strong evidence that the combination of HDACIs with other classes of drugs is preferential to potentiate the anticancer activity [34,35], as the mechanisms of action of HDACIs can make cancer cells more prone to other drugs [35], i.e., TKIs or immunotherapy. The current clinical investigation demonstrated that after the administration of HDACIs, the activity of kinases in the MAPK pathway, as well as the expression of immune modulatory cell surface proteins, are regulated differently [36].

In this study, we used our validated approach, therapeutically guided multidrug optimization, to rapidly optimize multidrug combinations specific to a selected phenotype (cell metabolism) [21]. This method, as compared to other drug optimization strategies, searches in only a few experimental steps along with in silico data modeling for low-dose multidrug combinations based on drug–drug interactions and activity [37,38,39,40]. Moreover, the concomitant drug testing in non-cancerous cells allowed direct elimination of possible toxicity.

We previously reported on the identification of an optimized four-drug combination specific to the human clear cell-renal cell carcinoma cell line 786-O [21]. The ‘one size fits all’ approach of cancer therapy, where patients are given a treatment based on their cancer type, a common genetic marker, or a mutant oncogene, remains unsatisfactory. Due to genetic and transcriptomic alterations, also present in cell lines, it becomes obvious why (patient-) cell-specific drug combinations are needed [21,22]. Not only varying in their origin, Caki-1 and 786-O cells are distinct from one another (Table S2). The cell cycle regulation protein p53 is normally expressed in Caki-1 cell but mutated in 786-O cells. In addition, where Caki-1 cells carry a wildtype *vhl* gene normally coordinating the expression of vascular endothelial and platelet-derived growth factor, 786-O cells carry a mutation and are therefore overexpressing both GF. Hence, this overexpression induces an upregulation of the related GFR and enhanced signaling to promote angiogenesis.

Therefore, we used our approach to optimize the treatment specifically to the individual need, reflected by the phenotype. In the first instance, the Caki-1-specific ODC contained panobinostat, vorinostat, and axitinib. Because of the oncogenic mutations and other cell-specific properties, whose interplay intrinsically guided the selection, the strongest anticancer activity was obtained by this drug combination containing two HDACIs and one TKI inhibitor.

Another critical point in the ccRCC treatment management is acquired resistance to sunitinib [41,42,43,44]. The induction of Caki-1-SR clones was performed both with low (1 µM) or high (10 µM) concentrations of sunitinib, in order to reflect intrinsic or acquired resistance. The doses correspond to the levels of sunitinib in the plasma of the patients or accumulated in a tumor, respectively [45]. Through fluorescence imaging, we were able to visualize the sequestration of sunitinib in lysosomal vesicles, being one pathway of drug resistance [41]. On the molecular level, proteins of the HIF-family and mTOR play a role in promoting sunitinib resistance [41,46]. Our ODC proved to be active not only in Caki-1 sunitinib-naive cells but also in three distinct sunitinib-resistant clones of Caki-1 cells (aforementioned) (Figure 2b and Figure 5c). As the signal transduction of certain signaling pathways and the activity of HDAC are not blocked by sunitinib, the ODC remains active in the sunitinib-resistant Caki-1 cells. As axitinib and pictilisib molecules are blocked being upstream of mTOR, hence it seems that sunitinib-induced activation of mTOR can be ruled out.

Our data show that our drug optimization performed in a simple 2-D in vitro cell culture can be translated to more complex homo- and heterotypic co-cultures, revealing different potential mechanisms of action (Figure 3 and Figure 5). The ODC presents a strong efficacy to inhibit cancer cell migration (Figure 3), hindering the uncontrolled spread of the Caki-1 cells. In addition, we observed alterations of cell cycle profiles (Figure 2 and Figure S11). Through this system, we were able to determine that the presence of fibroblasts influences the treatment response. Further studies will be needed to elucidate the role of other cell types present in a tumor lesion, i.e., immune cells as it has been demonstrated that HDACIs can regulate the expression of immune-modulatory cell surface proteins as well as the activity of immune cells [16,47].

To gain more insight into the possible response of Caki-1 cells to the mixture of panobinostat, vorinostat, and axitinib, we performed RNA sequencing analysis of untreated Caki-1 cells to determine important drug targets. It showed a high expression of transcripts related to cell adherence and motility (Table S6), which is in line with our results demonstrating the migrating capacity of 3-Dc cultured in the presence of 0.5 mg/mL collagen (Figure 3). The RNA sequencing of untreated Caki-1 cells unraveled not only a set of highly expressed genes that encode proteins involved in cell attachment but also involved in protein translation or cellular homeostasis (Table S6). Interestingly, the drugs composing the ODC influence these processes directly, as (i) panobinostat inhibits the attachment and thereby the motility of cancer cells [48,49], (ii) vorinostat blocks the translation machinery [50,51], or (iii) axitinib alters the cellular homeostasis by inducing the permeabilization of mitochondria [52].

Phosphoproteomics analysis of Caki-1 cells, with a focus on protein tyrosine kinases, showed that different kinases are phosphorylated, hence active in Caki-1 that are targets for inhibition by TKIs. In order to look for further effect amplification, we coupled pTyr-based phosphoproteomics to INKA activity analysis [53] and checked if the untreated cell line phosphorylation profile affecting tyrosine kinases might justify the addition of another compound to the mixture. In Figure 4b, the top 20 INKA-based most active kinases in Caki-1 cells are presented, including MET, EGFR, SRC, and PTK2. It is known that activation of these receptor tyrosine kinases stimulates the RAS–RAF–MEK–MAPK and the PI3K–AKT pathways, in turn promoting cell growth and angiogenesis [54,55,56,57,58]. Moreover, MET activation is already recognized in (cc)RCC and linked to the VEGF(R) inhibition resistance [59,60,61,62].

As tyrosine phosphorylated proteins constitute just a small percentage of the phosphoproteome, only a partial image can be seen of the disease network of Caki-1 based on these data exclusively. Interestingly, despite the high activation of EGFR in untreated Caki-1 cells, the EGFR-targeting drug, erlotinib, present in the initial drug set, was eliminated from the drug optimization process due to unfavorable drug–drug interactions. This could be explained by the presence of HDACI in the optimization process and (epi)genetic modulations influencing drug–drug interactions. We chose to add pictilisib or saracatinib to the originally optimized ODC, targeting directly at the level of PI3K and Src upstream of the MAPK pathway, based on targeting complementary components of oncogenic signaling.

Although the ODC targets several proteins highlighted through the RNA sequencing and phosphoproteomic data, the anticancer efficacy of the ODC was reduced in 3-Dc and 3-Dcc (Figure 5). We assume that through the apolar matrix-containing environment and the strong cell-to-cell connection in the spheroid, the penetration of the drugs is decreased. Through intracellular crosstalk, downregulation of receptor expression, and altering cell signaling pathways, the cells start to protect themselves towards the treatment. Especially in the presence of fibroblasts (3-Dcc), this loss of efficacy is more potent. This is an important observation, because the ODC in the supporting fibroblasts, cultured in 2-D, was mostly inactive. This implies that drug combinations need to be established taking into account that the targets change in accordance with the tumor composition and its protective barriers.

Remarkably, after the addition of either pictilisib or saracatinib, the sensitivity to this four-drug ODC was enhanced in 3-Dc as well as in 3-Dcc when fibroblasts were present (Figure 5) [63,64,65]. Further, in all cases, the ODC containing the fourth drug, pictilisib, was active and synergistic in all the sunitinib-resistant Caki-1 clones (Figure 5).

In our study, the combination of the ODC with pictilisib was more effective than the addition of saracatinib. Research by Hirsch et al. has demonstrated that kinases of the Src family can be targets of HDACI, augmenting the anticancer activity [66]. Further, it has been demonstrated that direct blockade of the Src kinase SRC-3 enhanced the anticancer activity of SAHA [67], and a similar result was obtained in our experiments through the combination of SAHA and saracatinib (Figure 5c). Nevertheless, the additional blockade of PI3K is more dominant than the blockade of Src, which relates to the role of potent MET and MAPK signaling as visualized by RNA sequencing and phosphoproteomics.

In the clinical investigation, the drugs belonging to both drug classes cause side effects at administered clinically doses (Table S8), increasing the possibility to bind to off-targets [16]. To reduce the probability of off-target toxicity, the ODC has been designed and optimized, a dose of a drug that is significantly (≥10-fold) lower than the dose in the clinical settings (Table S3). Tested doses of the drugs interact synergistically, which enhances their anticancer activity while reducing the side effects. Based on our results, we are aware that the activity and synergy of the ODC on cell proliferation and migration is mostly due to the activity of panobinostat. This can be a limiting factor of translation to treat cancers that are insensitive to panobinostat. However, through the combined use with drugs from two different drug classes, the treatment response might be restored.

In this study, we did not investigate the full mechanism of action of the ODC and we did not verify the blockade of the cognate targets, but our data indicated that the selectivity to cancer cells (Figure 2), inhibition of migration (Figure 3), and induction of cell death (Figure 2) are caused through obstruction of the main targets. Dose reduction directly correlates to increased binding of high-affinity targets only, which increases the efficacy of the applied drugs [68]. As we were able to reduce the doses of vorinostat and axitinib by 10-fold, we expect that off-target effects can be reduced while blocking the relevant targets.

Using the TGMO-based screen, we were able to develop the ODC to be specific to a defined phenotypic appearance in response to single and combined drug treatment. This treatment response can be based on a specific genetic or physiological background. Thereby, we take into account that each patient is different. Nevertheless, further elucidation of the genetic background and its influence on the treatment sensitivity will be needed to determine the match between patient and treatment options. Although we performed RNA sequencing and phosphoproteomics, more in-depth analysis is required to determine potential biomarkers simplifying the choice of medication. To find potential biomarkers and to invigorate the benefit of our ODC, it is necessary to investigate the anticancer effect on patient material and in vivo.

HDACIs, being versatile regarding their activity, enhance the efficacy of TKIs and of immune-modulatory agents (IMAs) [16,47,68]. Due to the versatile application and based on pre-clinical evidence, clinical trials have been launched (Table S7) combining vorinostat with pembrolizumab (NCT02619253) and enantiostat with aldesleukin (interleukin 2; ClinicalTrials.gov Identifier: NCT01038778 [69]) for the treatment of RCC. Both trials were designed to determine the response rate and safety profile of the combination treatment and the patient enrolment was based on the treatment history and the histology of the cancer [69]. By combining HDACIs and IMAs, synergistic interactions were observed, increasing the susceptibility of the tumor cells to either of the two drug classes. Therefore, the safety/toxicity profile was ameliorated and in the case of administration of enantiostat with aldesleukin, an overall survival of 65.3 months was observed [69].

Finally, our Caki-1-specific ODC presented an anti-angiogenic effect via the reduction of endothelial cell metabolism (viability) but not via endothelial cell migration [70,71]. The comprehensive angiostatic effect was confirmed in the CAM model (see Figure S11), a known in vivo model to study angiogenesis [72,73]. This effect can result because through HDACIs, the transcription factor HIF-1α can become hyperacetylated, leading to the inhibition of angiogenesis [2]. This additional mechanism of action of the ODC may lead to a more pronounced overall anticancer effect. This is of particular interest in RCC, known to be chemo- and radiotherapy resistant, but relatively well treated with anti-angiogenic drugs [61,62,74] unless acquired drug resistance appears in patients to these angiostatic drugs [2,75,76]. As we previously reported [77], epigenetic drugs in combination with targeted angiogenesis inhibitors showed prolonged activity both in pre-clinical models and in vivo trials [21,77], which confirms our findings.

Summarizing, this study showed that our TGMO method selected the cell line-specific drug combination composed of HDACIs and TKIs. This ODC was active in complex 3-D heterotypic in vitro models. Ideally, similar analyses should be performed using cells from freshly resected RCC patient material to demonstrate the superior value of combined targeted therapy in the personalized clinical management of RCC patients. As shown in recently published studies on treatment optimization for colorectal carcinoma [78], we intend to pursue this line of investigation in the near future for RCC management. Taken together, our strategy applies well in vitro and can be considered a powerful tool for the design of phenotype-specific individualized treatments for cancer.

## 4. Materials and Methods

### 4.1. Cell Culture

Caki-1, human renal cell carcinoma cell line, as well as HEK-293T, a human embryonic non-cancerous cell line, were generously donated by Dr. Dormond (Department of Visceral Surgery, CHUV, Lausanne, Switzerland). Caki-1 cells were cultured in DMEM medium and HEK-293T in RPMI medium supplemented with 10% fetal bovine serum (Biowest, Nuaillé, France, S1810-500) and 1% penicillin/streptomycin (Bioconcept, Basel, Switzerland, 4-01F00-H) in a humidified incubator with 5% CO_2_ at 37 °C. ECRF24 cells [21,65] were genuinely donated by Prof. Arjan W. Griffioen (Department of Medical Oncology, Vrije Universiteit Amsterdam, The Netherlands). ECRF24 cells were cultured in 0.2% gelatin-coated flasks in a 1:1 mixture of DMEM and RPMI medium supplemented with 10% fetal bovine serum and 1% penicillin/streptomycin. NHDFα cells were kept in a special fibroblast medium and supplements purchased from Vitaris (Baar, Switzerland, C-23120-PRO). Cells were tested frequently for mycoplasma contamination and were authenticated by Microsynth AG (Balgach, St. Gallen, Switzerland). Through STR systems from Promega (Zurich, Switzerland) and database comparison, the cell line identity was analyzed and confirmed.

### 4.2. Sunitinib-Resistant Caki-1 Clones

Sunitinib resistance (-SR) in Caki-1 cells was induced through three different techniques to obtain three diverse cellular clones with an intrinsic or acquired resistance (Table S2). (i) The first clone was prepared by applying continuously increasing doses of sunitinib through the course of chronic treatment with every cell split. (ii) The Caki-1 cells were treated chronically with 1 µM of sunitinib for >8 weeks. (iii) A dose of 10 µM sunitinib was applied once for 72 h and afterward exchanged through medium to promote the proliferation of the cells that survived the treatment period.

### 4.3. Small Molecule-Based Drugs

All compounds (Table S1) were dissolved in sterile DMSO (Sigma-Aldrich (Merck, Darmstadt, Germany), D8418-50ML). Tacedinaline (20 mg/mL), panobinostat (5 mg/mL), vorinostat (5 mg/mL), tubacin (5 mg/mL), axitinib (20 mg/mL), erlotinib (15 mg/mL), dactolisib (1 mg/mL), dasatinib (5 mg/mL), tozasertib (20 mg/mL), and sorafenib (40 mg/mL) were purchased from LC Labs (Woburn, MA, USA) or from Selleck Chemicals (Houston, TX, USA) [21]. Pictilisib (30 mg/mL) and saracatinib (20 mg/mL) were purchased from Selleck Chemicals. A maximal concentration of 0.05% DMSO was allowed for any of the screened conditions and was used as a control (CTRL). Sunitinib was donated by the University Hospital of Geneva (HUG) and applied at a concentration of 1, 10, or 20 µM as a positive control.

### 4.4. Combinatorial Drug Screen and TGMO-Based Screen

The inhibition of the cell metabolic activity was selected as a target phenotype and was measured to analyze the activity of each drug combination using the CellTiter-Glo solution (Promega, G7572). The therapeutic window was determined by comparing the efficacy of each drug combination between cancerous RCC and non-cancerous cell lines. Second-order step-wise linear regression models using Matlab^®^ (https://www.mathworks.com/products/matlab.html) were applied to model the results as described by Weiss et al. [39]. Statistical measures—Cook’s distance, regression coefficient, root mean square error, and data point correlations (Figure S3)—defined the accuracy and reliability of regression models for each iteration [79], with an ANOVA lack of fit test confirming the selection of a relevant model structure. After three sequential search rounds, the compounds alisertib, crenolanib, and icaritin were excluded from further experiments based on the regression model elimination. More detailed information can be found in the Text S1.

### 4.5. Migration Assays

A scratch assay [80] was performed to quantify the migration of RCC (Caki-1) and endothelial (ECRF24) cells after 5 and 7 h after exposition to the treatment. With a sterile scratch tool (Peira Scientific Instruments, Beerse, Belgium), a scratch was placed following immediate treatment administration. To image in a time-dependent fashion, a Leica DMI3000 microscope (Leica, Rijswijk, The Netherlands) at ×5 magnification and to analyze the Universal Grab 6.3 software (DCILabs, Keerbergen, Belgium) were used. Images were taken at T = 0 h and continuously until T = 7 h. Using the Scratch Assay 6.2 (DCILabs) software, the broadness of the scratch was quantified automatically and the absolute wound closure calculated (initial minus final scratch surface) [81].

### 4.6. 3-D Homo- and Heterotypic Spheroid Cultures

To obtain spheroidal cultures, 3000 Caki-1 cells were seeded in round-bottom 96-well low attachment plates (Greiner Bio-One, Frickenhausen, Germany, Cellstar^®^ 650970) in DMEM medium supplemented with 5% FBS and 2.5% Matrigel (Corning, Root, Lucerne, Switzerland, matrigel #354230). To promote migration, the culture medium was supplemented with 0.5 mg/mL collagen type 1 from rat tail (Sigma-Aldrich, C3867) instead of Matrigel. Different rigidity of the milieu was obtained by adding varying concentrations of Matrigel (0%, 2.5%, or 25%).

### 4.7. Cell Cycle Distribution and Cell Death Analysis

Cell cycle distribution was discriminated by staining a cell pellet of approximately 5 × 10^5^ cells with 500 µL FxCycleTM PI/RNase staining solution (Invitrogen, Carlsbad, CA, USA, F10797). Samples were analyzed on an Attune NxT flow cytometer (Thermo Fisher Scientific/Invitrogen, Basel, Switzerland).

### 4.8. INKA Analysis of Phosphoproteomic Data

The Integrative Inferred Kinase Activity (INKA) analysis was performed as described before [53,82]; see Text S3 for details. Proteomics data were deposited in ProteomeXchange via the PRIDE repository [82] with accession number PXD016475.

### 4.9. Chorioallantoic Membrane (CAM) of the Chicken Embryo

The chorioallantoic membrane model was used to determine the anti-angiogenic activity of both ODCs and the monotherapies in vivo model [21,72]. Fertilized chicken eggs were incubated in a hatching incubator at 37 °C with a relative humidity of 65% [83]. Eggs were incubated in a stationary position until EDD 7, the start of experimentation. A plastic ring was deposited on the CAM membrane and 20 μL of treatment was administered within the ring. Treatment was performed twice, on EDD 7 and 8, and the membranes were imaged via fluorescence angiograms on EDD 9 using an epi-fluorescence microscope (Nikon AG, Eclipse FN1, Minato-ku, 108-6290 Tokyo, Japan) coupled to a pco.pixelfly camera (Figure S11). Fluorescein isothiocyanate dextran (FITC-dextran, 20 kDa, 25 mg/mL, Sigma-Aldrich) was injected intravascularly. To increase vascular contrast, 50 μL of black ink were injected (Pelikan, Witzikon, Switzerland) into the embryonic cavity. Image-based quantification using the number of branching points/mm^2^ was performed using our ImageJ-based software [84].

### 4.10. Statistical Analysis

The data is presented as the mean of multiple independent experiments. Error bars represent the standard error unless otherwise specified. Significance was determined depending on the experimental setting using either the two- or one-way ANOVA test with posthoc Tukey’s multiple comparison test or a student’s *t*-test (Graphpad Prism^7^). Statistically significant values were calculated vs. the CTRL or single regimens, and p-values are specifically indicated in each figure legend and marked with *** (*p* ≤ 0.001), ** (*p* ≤ 0.01), or * (*p* ≤ 0.05) in the related graphs.

## 5. Conclusions

Our results demonstrate that the optimized low-dose multidrug combination of two HDACIs and one TKI is highly effective in in vitro settings inhibiting the migration of Caki-1 cells, potentially reverting the invasive and metastatic phenotype. Functional analysis revealed the induction of cell death and the potency to overcome acquired sunitinib resistance. Through the addition of a second TKI, the anticancer activity of the ODC can be enhanced, facilitating a reduction of the metabolic activity of sunitinib-naïve and sunitinib-resistant Caki-1 cells cultured in 3-D. Further mechanistic studies and preclinical validation are needed to reveal the potency of the optimized drug combinations in more complex models.

## Supplementary Materials

The following are available online at https://www.mdpi.com/2072-6694/12/11/3172/s1, Text S1: TGMO-based screen and optimization process; Text S2: RNA sequencing; Text S3: INKA analysis of phosphoproteomics; Figure S1: Initial drug set containing four HDACI, four TKI and two serine-threonine kinase inhibitor used in the TGMO-based drug optimization; Figure S2: Drug response curves for an initial drug set of four HDACI, four TKI and two serine-threonine kinase inhibitor used in the TGMO-based drug optimization; Figure S3: Linear regression models to interpret the TGMO-based search; Figure S4: Graphs accompanying the calculation of the Combination Index; Figure S5: Characterization of chronically sunitinib-treated cell clones; Figure S6: Efficacy of non-dose-optimized drug combinations screened in cancerous and non-cancerous cell lines; Figure S7: Inhibition of the proliferation of Caki-1 cells; Figure S8: Translation of the multi-drug combination in 3D cultured Caki-1 spheroids indicated through the cell viability and the inhibition of cell migration; Figure S9: Drug-drug interactions measured in 2D cultured Caki-1 cells; Figure S10: Drug-drug interactions measured in 3D cultured Caki-1 cells; Figure S11: Anti-angiogenic activity of the ODC in vitro and in vivo; Table S1. Information on all drugs used for the TGMO-based screen; Table S2. Genetic distinction between cell lines and the description of –SR cells; Table S3. Doses in clinical use for panobinostat (pan), vorinostat (vor), axitinib (axi), pictilisib (pic) and saracatinib (sar); Table S4. Combinatorial Index of three drug combination panobinostat (pan), vorinostat (vor), axitinib (axi) at various doses; Table S5. Cross-validation of ODC in ccRCC and non-cancerous cell lines doses; Table S6. 35 highest expressed transcripts of Caki-1 cells validated through RNA sequencing; Table S7. RNA expression of saracatinib (sar), pictilisib (pic), axitinib (axi) or crizotinib (cri) drug targets in Caki-1 and Caki-1-SR clone 1 cells.; Table S8. HDACI combined with immune -modulatory regimens in clinical trials.; Table S9. Side effects of panobinostat (pan), vorinostat (vor), axitinib (axi) and pictilisib (pic) reported in the cardiovascular, central nervous, endocrine and gastrointestinal system and potential cross activities. References [85,86,87,88,89,90,91,92,93,94,95,96,97,98,99,100,101,102,103,104,105,106,107] are cited in the Supplementary File.
