# Supplementary material for: Optimized Combination of HDACI and TKI Efficiently Inhibits Metabolic Activity in Renal Cell Carcinoma and Overcomes Sunitinib Resistance
Source: Cancers (Basel). 2020 Oct 28;12(11):3172. doi: 10.3390/cancers12113172 (PMC7693411; doi:10.3390/cancers12113172)
Supplement: Supplementary file 1 [file cancers-12-03172-s001.pdf]

Supplementary Materials: Optimized Combination of HDACI and TKI Efficiently Inhibits Metabolic Activity in Renal Cell Carcinoma and Overcomes Sunitinib Resistance

Magdalena Rausch, Andrea Weiss, Marloes Zoetemelk, Sander R. Piersma, Connie R. Jimenez, Judy R. van Beijnum and Patrycja Nowak-Sliwinska

Supplementary Information

Text S1: TGMO-Based Screen and Optimization Process

We used the Therapeutically Guided Multidrug Optimization (TGMO) method [22,21] to describe the drug-drug interactions between the set of 10 drugs (Figure S1) at the two doses used (ED_{20} and ED_{10}) at the beginning of the search. TGMO method allowed to select the final optimized multidrug combination (ODC) consisting of panobinostat, vorinostat and axitinib; see Figure 1.

The optimization is based on the orthogonal array composite design (OACD) matrices. Each matrix was specifically designed to obtain the optimal and maximal information of drug combinations performed in each search. In Search 1 we tested 10 drugs, from which three were excluded for another search. From the remaining 7 drugs another three were excluded in Search 2, to finally validate in Search 3, which four-drug combination would be the most effective [38,39].

More detailed, the matrix consists of three parts: (i) to expose the linear effects of the drugs demonstrating single and two-drug interactions as estimated regression coefficients, (ii) to investigate linear and quadratic effects, as well as to inform on the non-linear response surface over multiple doses, (iii) to define the most influential variables (a resolution IV matrix [79]).

The first step of the optimization is to perform drug dose-response curves and define the drug dose input for each of the 10 drugs, in our case the ED_{20} and ED_{10}. Afterward, throughout the three searches, drug interactions and dose effects are eliminated to guide through the selection process. As only a small portion of possible combinations is tested experimentally the remaining combinations and their efficacies can be modeled mathematically through step-wise second-order linear regression analysis by Matlab®.

The three searches are performed on cancerous cells (Caki-1), but simultaneously on non-malignant embryonic kidney cells (HEK-293T) to determine the difference between the two. This difference is called the therapeutic window (TW), a secondary model to visualize the selectivity of the drug combination activity. Consequently, the most optimal effect is depicted as opposite regression coefficients for anti-cancer efficacy (negative) and the TW (positive).

Text S2: RNA Sequencing

Using an RNA easy® Plus kit (74134, Qiagen, Hilden, Germany) and following the manufacturer's instructions RNA of Caki-1 cells was extracted. We executed the RNA quality control with FastQC v.0.11.5, the library preparation using TruSeqHT Stranded mRNA (Illumina), and sequencing on an Illumina HiSeq 4000 System using 100-bp single-end reads protocol. Reads were mapped to the human genome (UCSC hg38) using STAR v.2.5.3a software with average alignment around 92%. PicardTools v.2.9.0 has been used to perform biological quality control and HTSeq v.0.9.1 to evaluate the raw counts. Normalization and differential expression analysis were performed with the R/Bioconductor package edgeR v.3.24.3 with calculating with a general linear model, negative binomial distribution, and quasi-likelihood F test.

Gene ontology enrichment analysis was performed in Enrichr (http://amp.pharm.mssm.edu/Enrichr) for biological process.

Text S3: INKA Analysis of Phosphoproteomic Data

Phosphoproteomics analysis of appropriate, non-treated, Caki-1 cells under study, was performed following established protocols and annotation pipelines [85,86]. Peptides were separated through nano liquid chromatography (Dionex U3000, Amsterdam, The Netherlands) on a Reprosil Pur (Dr. Maisch GMBH, Ammerbuch-Entringen, Germany) C18 column (40 cm × 75 µm) applying a 90 minute acetonitrile gradient (2–32% in 0.1% formic acid). The inject-to-inject time was 120 min. We determined the sequence of peptide chains on-line on a Q Exactive-HF Orbitrap mass spectrometer (Thermo Scientific, Bremen, Germany). After ionization at 2 kV, MS1 masses were measured at R = 70,000 (AGC 3E6) and MS2 masses at R = 15,000 (AGC 1E6, MaxIT 64 ms). Peptides charged > +1 were fragmented (isolation-width 1.4 Da) at NCE of 25 in a top-15 experiment. Dynamic exclusion time was 30 sec with a repeat-count of 1.

To identify phosphopeptides and phosphoproteins, MS/MS spectra were searched against Swissprot human proteome (cannonical_and_isoforms, downloaded February 2018, 42,258 entries) using MaxQuant 1.6.0.16. Enzyme specificity was set to trypsin and up to two missed cleavages were allowed. Cysteine carboxyamidomethylation (Cys, +57.021464 Da) was treated as fixed modification and serine, threonine, and tyrosine phosphorylation (+79.966330 Da), methionine oxidation (Met,+15.994915 Da) and *N*-terminal acetylation (*N*-terminal, +42.010565 Da) as variable modifications. Peptide precursor ions were searched with a maximum mass deviation of 4.5 ppm and fragment ions with a maximum mass deviation of 20 ppm. Peptide, protein, and site identifications were filtered at an FDR of 1% using the decoy database strategy. The minimal peptide length was 7 amino-acids and the minimum Andromeda score for modified peptides was 40 and the corresponding minimum delta score was 6 (default MaxQuant settings). Peptide identifications were propagated across samples with the match between runs option checked. Phosphopeptides were quantified by counting MS/MS spectra (spectral counts) or by their extracted ion intensities ('Intensity' in MaxQuant).

Integrative Inferred Kinase Activity (INKA) scores and associated networks were generated based on phosphokinase and phospho-substrate data as described [53] and presented with the outline of the top 20 active kinases (i.e. highest ranking INKA scores) of untreated samples. For interpretation and visualization of differential phosphoprotein expression, normalized count data were used.

Supplementary Figures

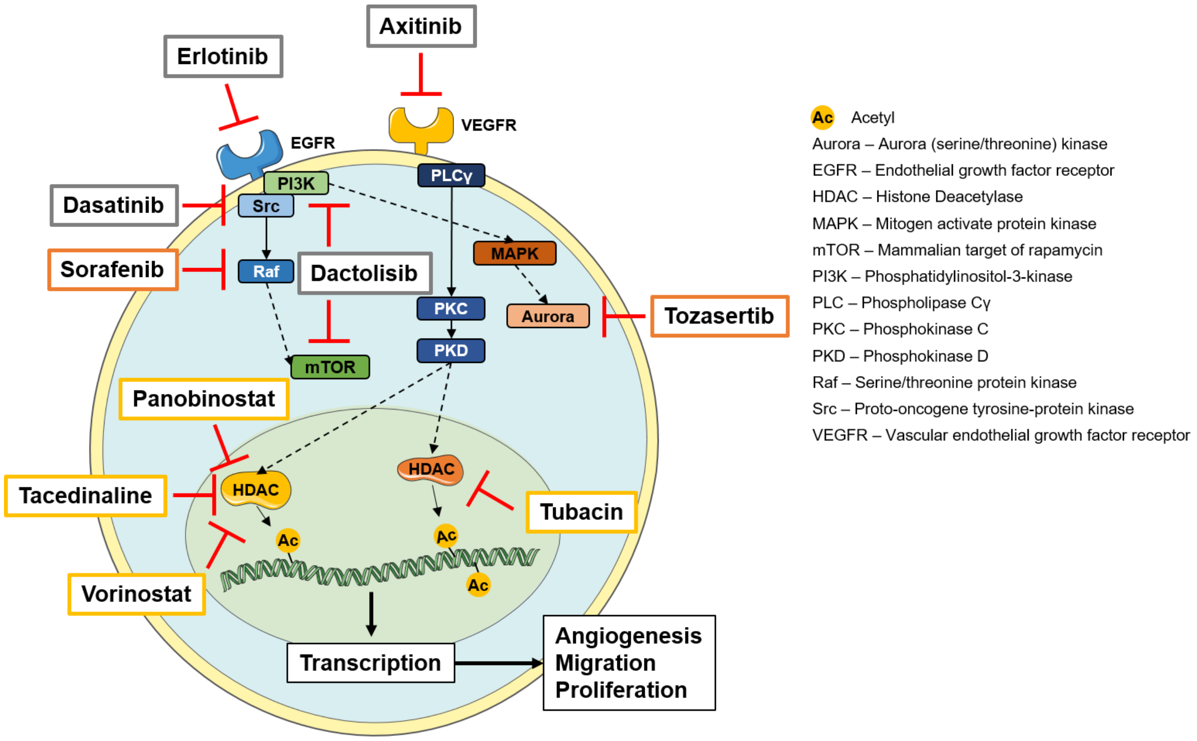

Figure S1: Initial drug set containing four HDACI, four TKI and two serine-threonine kinase inhibitor used in the TGMO-based drug optimization. Schematic representation of initial drug set and their upstream (extracellular receptors) or downstream targets (intracellular signaling proteins) in a cell. The four HDACI—tacedinaline, panobinostat, vorinostat and tubacin—are shown in yellow frames, the four TKI—axitinib, erlotinib, dactolisib and dasatinib—are presented in grey frames and the two serine/threonine kinase inhibitors—tozasertib and sorafenib—are highlighted in orange frames.

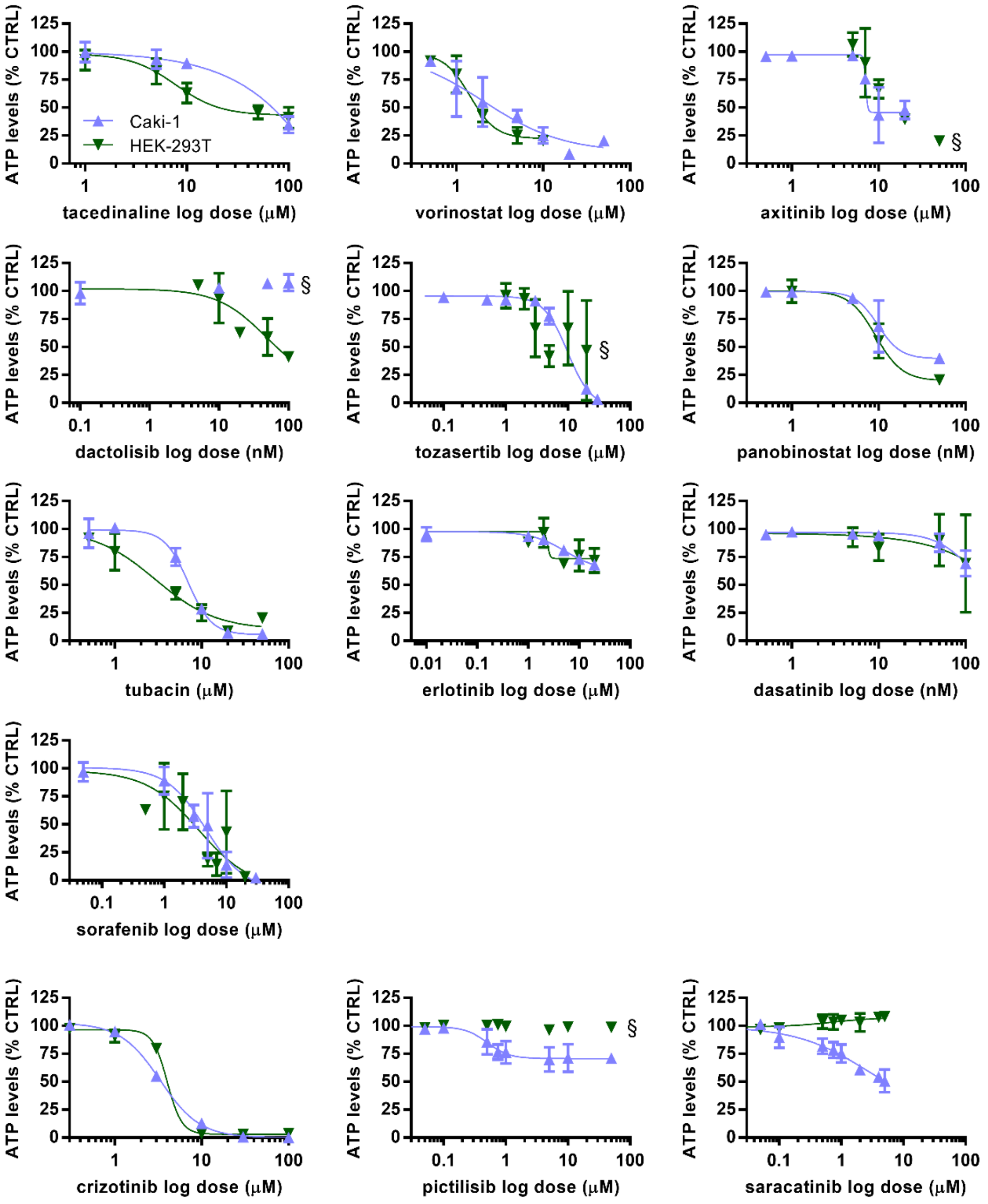

Figure S2: Drug response curves for an initial drug set of four HDACI, four TKI and two serine-threonine kinase inhibitor used in the TGMO-based drug optimization. Drug dose-response curves were performed in Caki-1 and HEK-293T cells for the initial set of 10 drugs (tacedinaline, vorinostat, axitinib, dactolisib, tozasertib, panobinostat, tubacin, erlotinib, dasatinib, sorafenib), as well as drugs included later in the study (crizotinib, pictilisib and saracatinib). A four-parameter non-linear fit was applied to the data using Graphpad Prism®. Ambiguous calculations are indicated with §. Error bars represent the SD (N = 3–5).

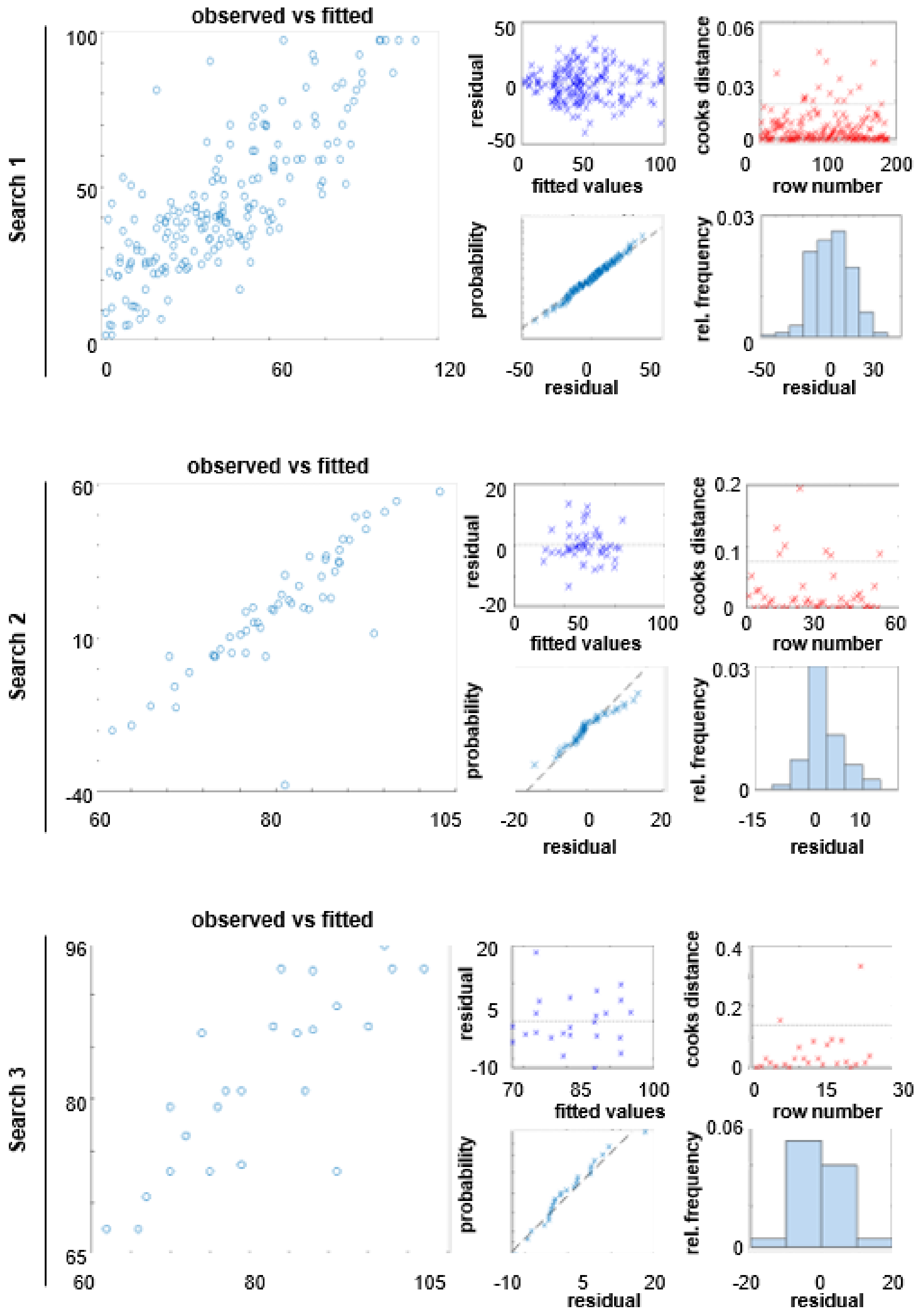

Figure S3: Linear regression models to interpret the TGMO-based search. Assessment of the accuracy and predictive value of the models through accompanying model analysis. The model analysis of all three searches, performed in Caki-1 cells. Observed vs. fitted values plot with the multiple determination (R2) (left plot), residual analysis plot of data to visualize constant variance (small graph top left), Cook's distance plot (small graph top right), Q-Q plot (small graph bottom left) and histogram of residuals (small graph bottom right).

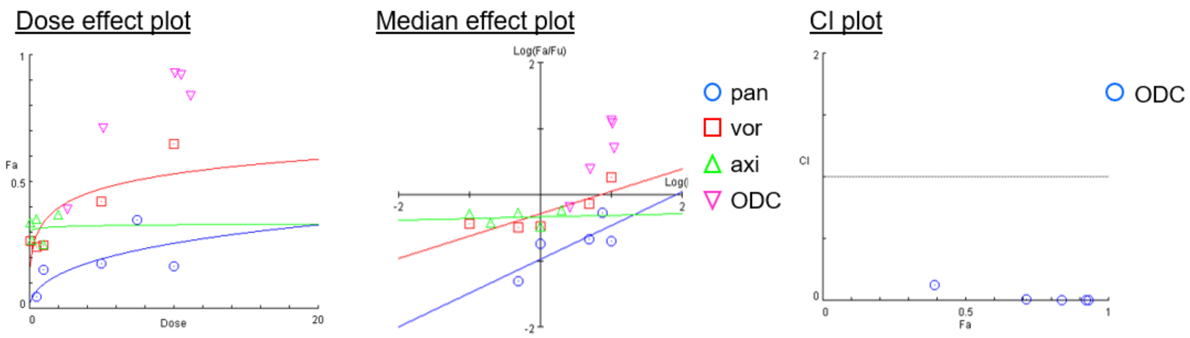

Figure S4: Graphs accompanying the calculation of the Combination Index. Isobolograms representing the dose- and median effect of panobinostat (pan), vorinostat (vor), axitinib (axi) and the three-drug combination (ODC) in Caki-1 cells. The combination index (CI) plot of the three-drug combination at different doses. Fiver points per condition have been used to draw the plots and to calculate the CI of the ODC.

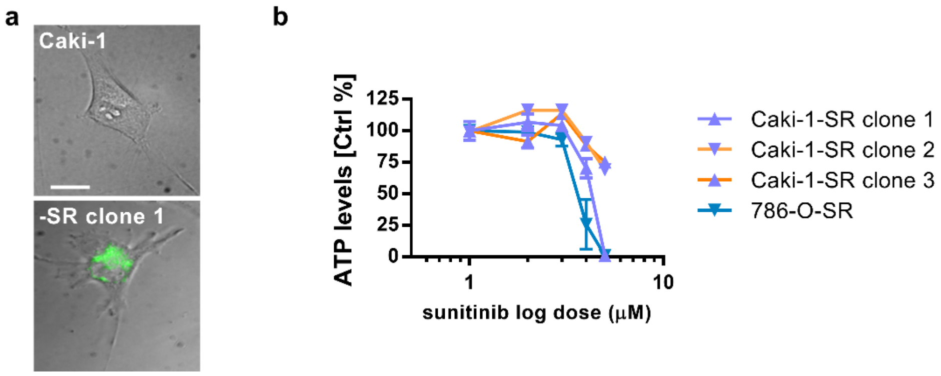

Figure S5: Characterization of chronically sunitinib-treated Caki-1 clones. (**a**) Representative picture of Caki-1 and Caki-1 sunitinib treated cells (Caki-1-SR clone 1). Accumulated sunitinib in lysosomal vesicles of Caki-1-SR clone 1 can be seen through its green-fluorescent signal. Scale bar represents 20 µm. (**b**) Dose-response curves for sunitinib in the four cells chronically treated with sunitinib. Error bars represent the SD ($N = 3$).

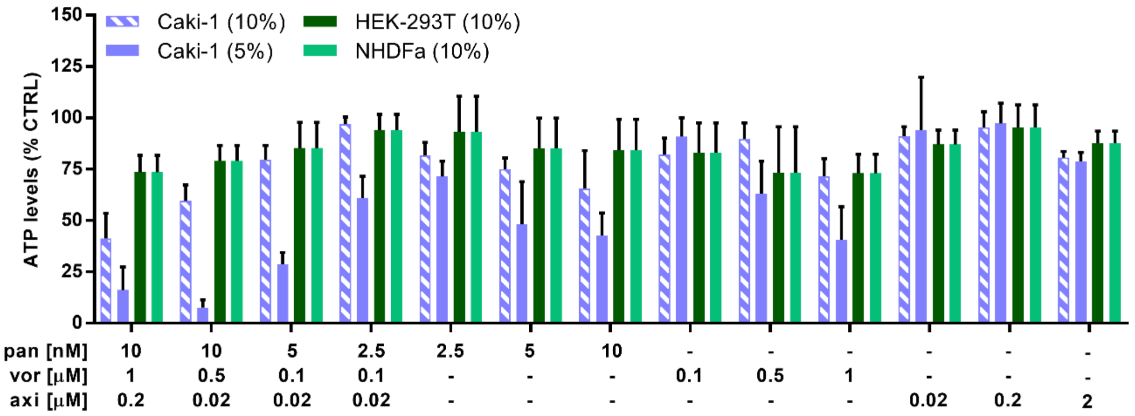

Figure S6: Efficacy of non-dose-optimized drug combinations screened in cancerous and non-cancerous cell lines. The efficacy on the ATP production measured in Caki-1, HEK-293T and NHDFα cells after 72 h treatment with non-optimized three-drug combinations Error bars represent the SD (*N* = 3).Statistical analysis revealed no significant changes between the represented conditions.

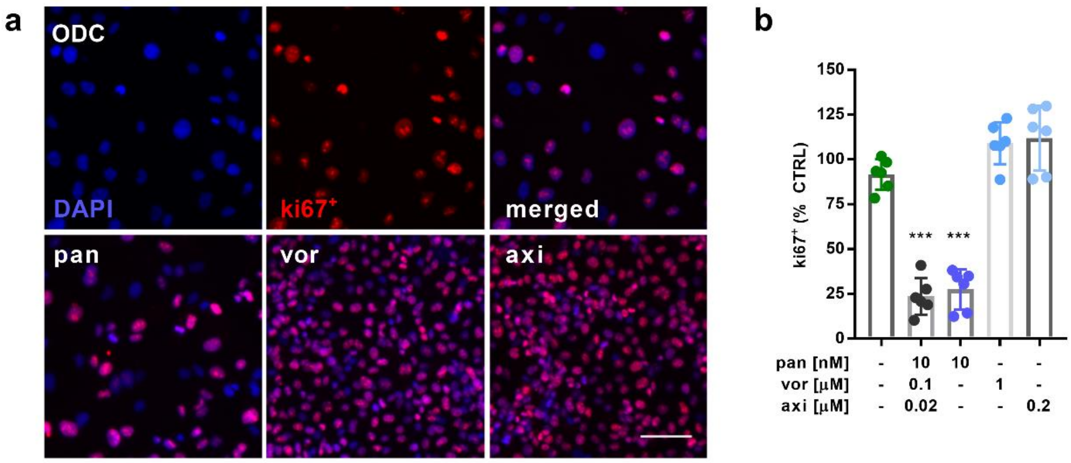

Figure S7: Inhibition of the proliferation of Caki-1 cells. (**a**) Representative images of DAPI (blue) and Ki67 (red) stained Caki-1 cells. Scale bar = 20 µm. (**b**) Bar graphs demonstrating the number of proliferating (Ki67^{+}) cells as percentage compared to the CTRL (*N* = 3). Error bars represent the SD and significance was determined with a one-way ANOVA and is represented with *** $p < 0.001$.

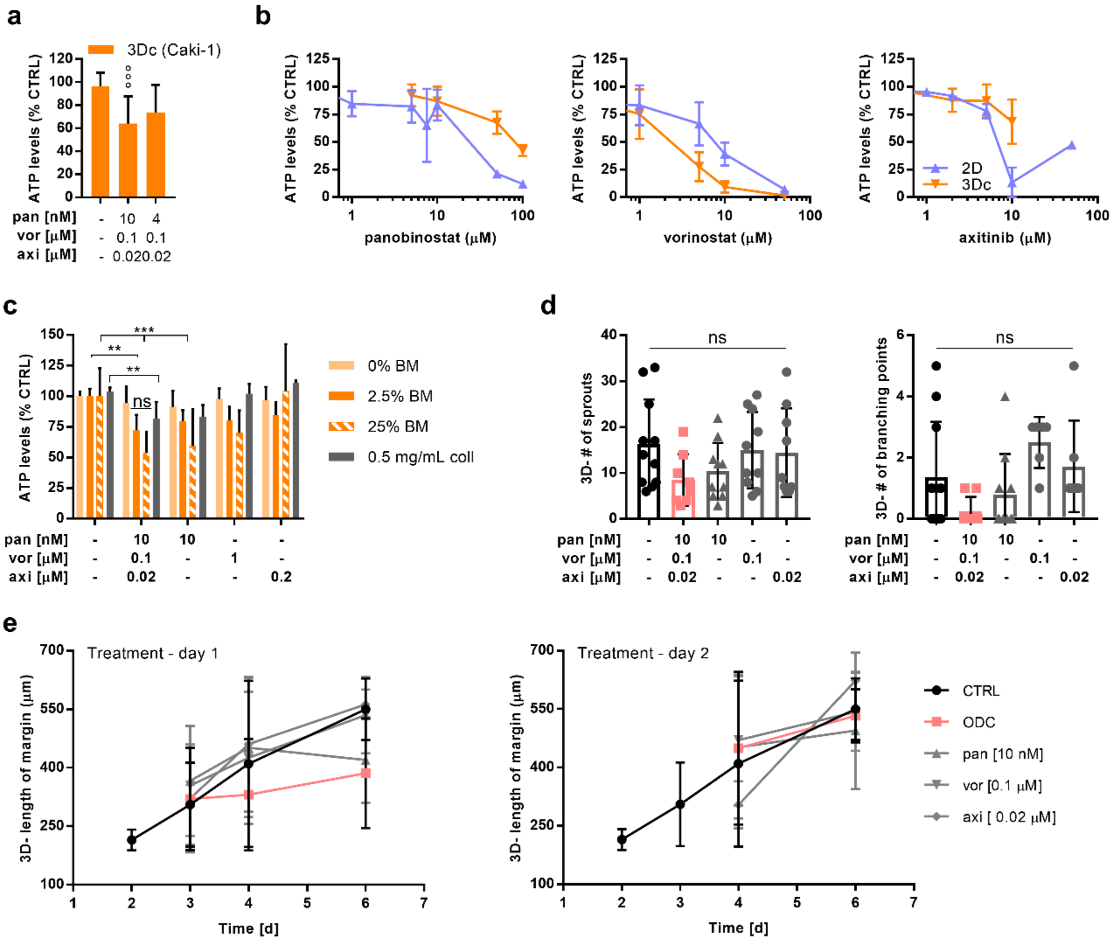

Figure S8: Translation of the multi-drug combination in 3D cultured Caki-1 spheroids indicated through the cell viability and the inhibition of cell migration. (**a**) Loss of anti-cancer activity of multi-drug combination applied at two different doses in 3Dc of Caki-1 cells. 1000 Caki-1 cells per well were seeded in low-attachment plates supplemented with 2.5% matrigel to promote the spheroid formation. (**b**) Dose response curves of panobinostat (pan), vorinostat (vor) and axitinib (axi) in 2D and 3Dc cultured Caki-1 cells. (**c**) Caki-1 3Dc in milieu with increasing rigidity by increasing the matrigel (BM) concentration and the addition of collagen (coll). (**d**) Analysis of the number of sprouts (left graph) and the number of branching points between sprouts (right graph) of 3Dc spheroids cultured in the presence of 0.5 mg/mL collagen. (**e**) Measurement of the length of the margin of Caki-1 spheroids cultured in 0.5 mg/mL collagen containing medium over a period of 6 days. Error bars represent the SD and significance was determined with a two- or a one-way ANOVA and is represented with °°° $p < 0.001$ compared to the CTRL only, ** $p < 0.01$ and *** $p < 0.001$ compared to the CTRL and the corresponding monotherapies ($N = 3$). ns, not significant.

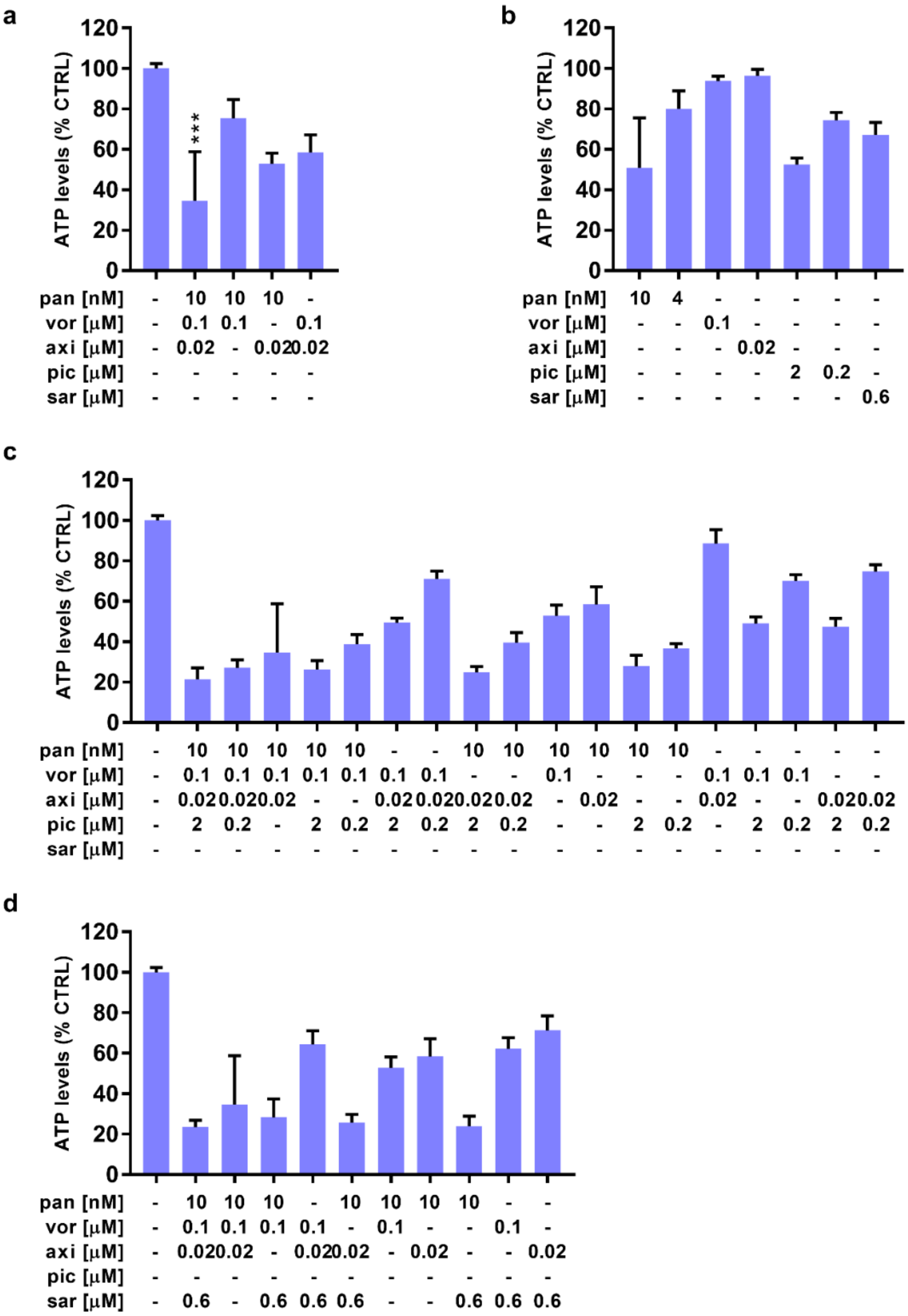

igure S9: Drug-drug interactions measured in 2D cultured Caki-1 cells**.** Validation of drug-drug interactions between the drugs panobinostat (pan), vorinostat (vor), axitinib (axi), pictilisib (pic) and saracatinib (sar) applied for 72 h on 2D cultured Caki-1 cells. (**a**) Original multidrug combination and two drug combinations; (**b**) Single drugs; (**c**) Four-, three- and two drug combinations with pic; (**d**) Four-, three- and two-drug combinations with sar. Error bars represent the SD and significance was determined with a one-way ANOVA and is represented with *** $p < 0.001$ calculated versus the CTRL and the corresponding monotherapies ($N = 3$).

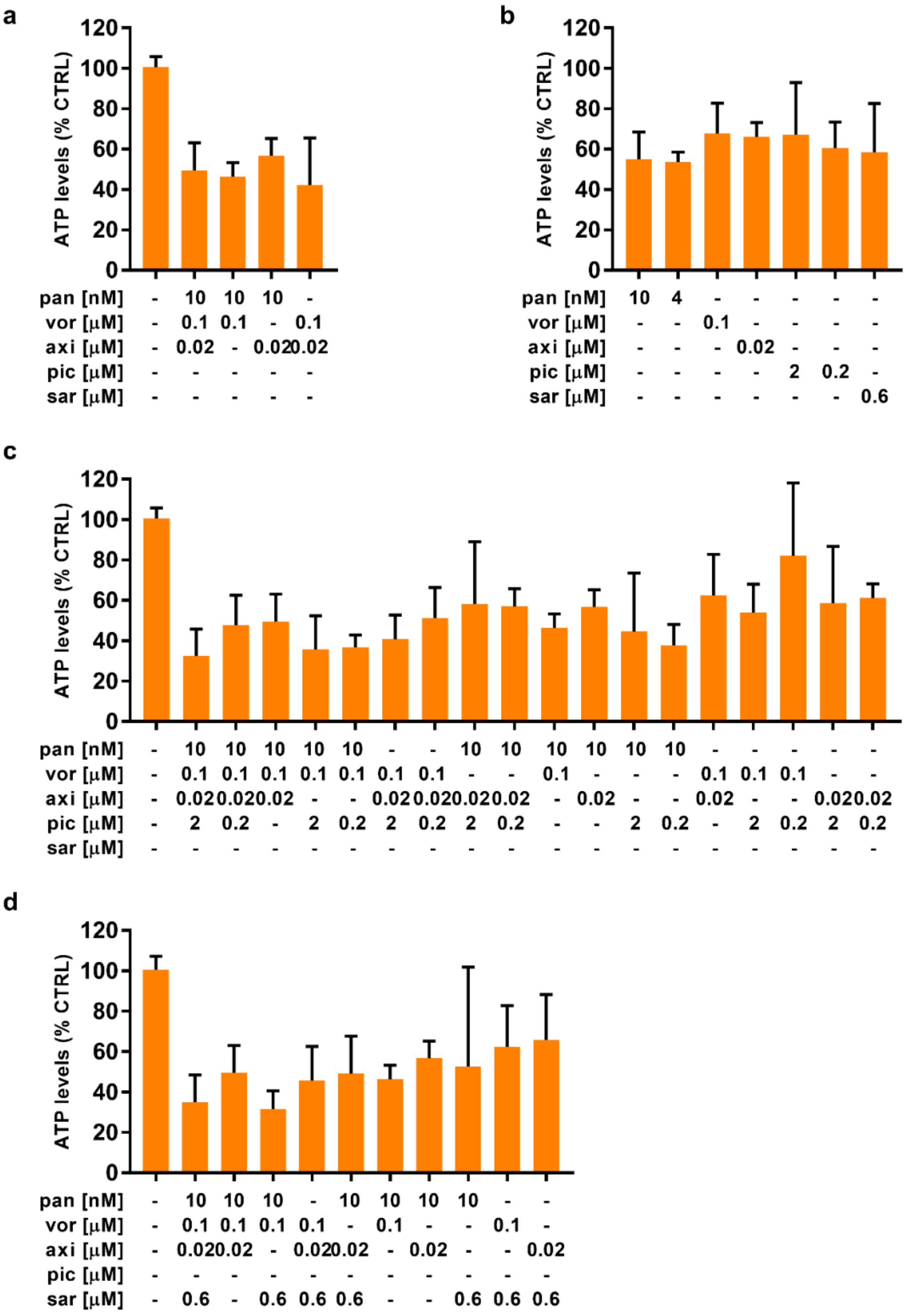

Figure S10: Drug-drug interactions measured in 3Dc Caki-1 cells. Validation of drug-drug interactions between the drugs pan, vor, axi, pic and sar applied for 72 h on 3Dc Caki-1 cells. (**a**) Original multi-drug combination and two drug combinations; (**b**) Single drugs; (**c**) Four-, three- and two drug combinations with pic; (**d**) Four-, three- and two-drug combinations with sar. Error bars represent the SD and significance was determined with a one-way ANOVA ($N = 3$). The presented conditions are not significantly different.

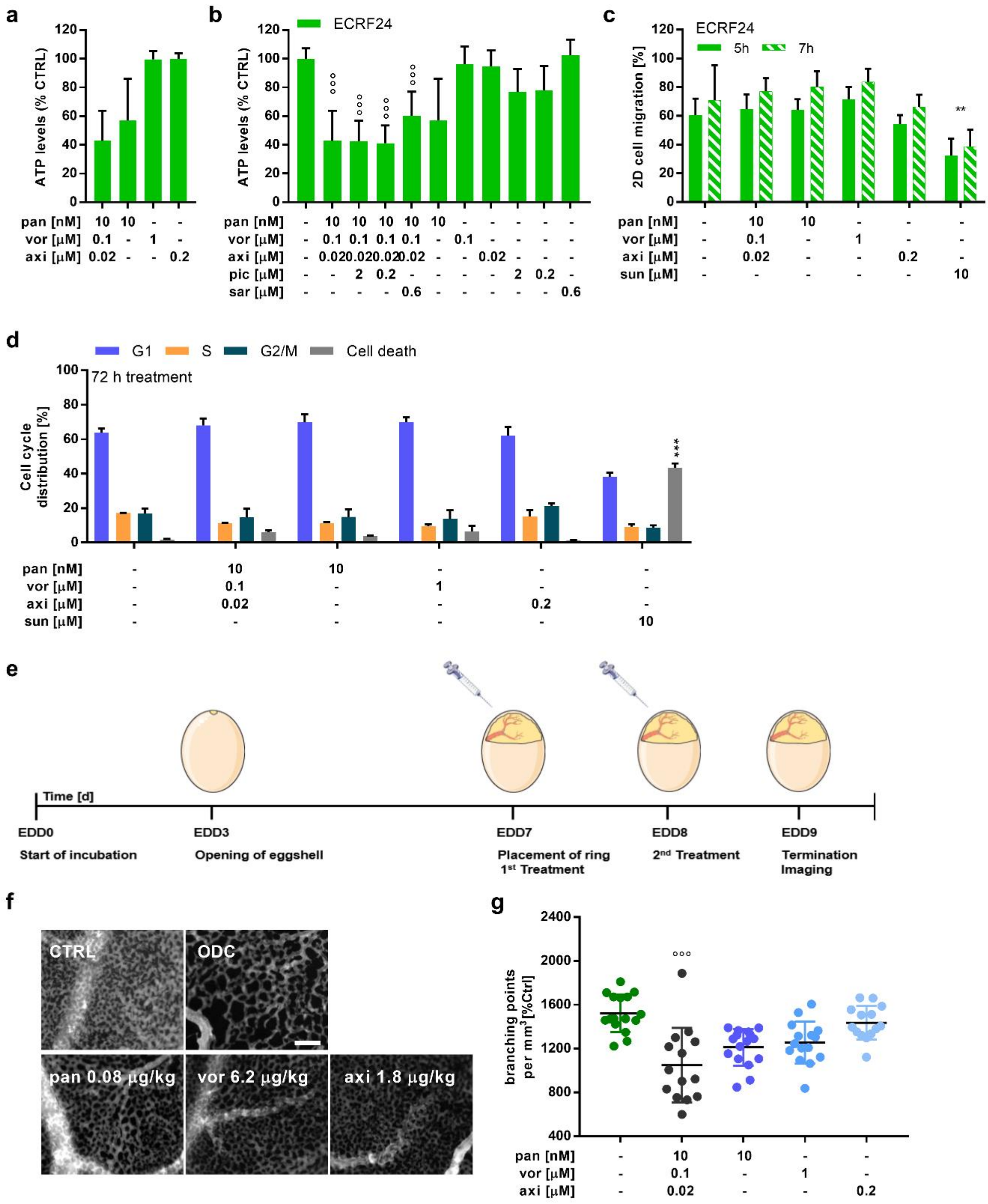

Figure S11: Anti-angiogenic activity of the ODC in vitro and in vivo. (**a**) Validation of the multi-drug combination and the corresponding monotherapies in ECRF24 cells. The treatment was applied with a medium supplemented with 10% foetal bovine serum. (*N* = 3) (**b**) Validation of the anti-cancer activity of the multi-drug combination supplemented with 2 µM and 0.2 µM pic or 0.6 µM. (*N* = 3) (**c**) Endothelial cell migration after 5 or 7 h of treatment with the multi-drug combination, its monotherapies, the positive (sun), and the negative CTRL. (*N* = 3) (**d**) Flow cytometry analysis with DNA binding propidium iodide (PI) after 72 h treatments presenting the cell cycle distribution within G1, S, G2/M phase, and cell death. (*N* = 3) (**e**) Timeline of the CAM in vivo experiments to analyze the anti-angiogenic activity of the multi-drug combination applied twice (EDD7 and EDD8). The eggs are placed in a humidified incubator on day 0 (EDD0). On EDD3 the eggshell is by making a small whole. Afterward, the eggs are re-incubated until EDD7, where the shell will be further opened to have access to the CAM its blood vessels. A ring is placed to apply the treatment and to perform the analysis in

the same position. The analysis is performed on EDD9; further information in the Materials and Methods section. (**f**) Representative images of the CAM vascularization in response to the treatments. The scale bar is 200 µm for all images. (**g**) Count of the number of vessel branching points/mm^3. (N = 6) Error bars represent the SD and significance was determined with a one-way ANOVA and is represented with * $p < 0.05$ and *** $p < 0.001$ versus the CTRL and the monotherapies. °°° $p < 0.001$ represents significance calculated versus the CTRL.

Supplementary Tables

Table S1. Information to all drugs used for the TGMO-based screen.

Compound	Abbrev.	Cellular Target	Original Indication	Development
Tacedinaline (CI-994)	tac	Class I HDACs	Advanced myeloma	Phase II*
Panobinostat (Farydak; LBH-589)	pan	Class I and II HDACs	Multiple myeloma	Approved*&**
Vorinostat (SAHA)	vor	Class I and II HDACs	Advanced Primary Cutaneous T-cell Lymphoma	Approved*
Tubacin	tub	HDAC6	Multiple myeloma, T cell lymphoma	Phase I*
Axitinib (Inlyta; AG013736)	axi	VEGFRs, PDGFR	Advanced renal cell carcinoma	Approved*&**
Erlotinib (Tarceva)	erl	EGFR	Non-Small-Cell Lung, Pancreatic Carcinoma	Approved*&**
Dactolisib (BEZ-235)	dac	mTOR	Advanced breast cancer	Phase I/II*&**
Dasatinib (Sprycel)	das	SRC	chronic myelogenous leukemia	Approved*&**
Tozasertib (VX-680)	toz	pan-Aurora Kinases	solid tumors and hematopoietic cancers	Phase I&II*&**
Sorafenib (Nexavar)	sor	VEGFRs, PDGFR	Hepatocellular and renal cell carcinoma	Approved*&**
Pictilisib (GDC-0941)	pic	PI3Kα/δ	Advanced or metastatic breast cancer [87]	Phase II*&**
Saracatinib (AZD-0530)	sar	Src, Bcr-Abl, Lck	Metastatic melanoma [88]; Metastatic head and neck squamous cell carcinoma [89]; T-cell acute lymphoblastic leukemia [90]	Phase II*&**
Crizotinib (Xalcori)	cri	c-MET, ALK	non-small cell lung carcinoma [91]	Approved*

Sunitinib (Sutent; SU11248)	sun	PDGFR	Renal cell carcinoma [43]	Approved*&**

Abbrev. = Abbreviation; *by the FDA, **by the EMA.

Table S2. Genetic distinction between cell lines and the description of –SR cells.

Cells	Origin	Differentially Expressed Genes					
**ccRCC**		**VhL**	**p53**	**hMSH2**	**hMLH1**	**MYC**	**PDGFRβ**
Caki-1	skin metastasis	wt	wt	wt	wt	overexpressed	wt
786-O	primary tumor	mut	mut	wt	wt	overexpressed	mut
HEK-293T	embryonic kidney	wt	mut	wt	mut	wt	wt
ECRF24	endothelium	NA	wt	NA	NA	NA	wt*
NHDFα	dermal fibroblast	NA	wt	NA	NA	NA	wt**
Caki-1	-SR Clone 1	treatment with increasing dose of sunitinib and maintenance at 1 μM [92]					
Caki-1	-SR Clone 2	chronic treatment with 1 μM sunitinib [42]					
Caki-1	-SR Clone 3	treatment with 10 μM sunitinib once for 72 h					
786-O	-SR	treatment with increasing dose of sunitinib and maintenance at 1 μM [92]					

ccRCC = clear cell renal cell carcinoma; VhL = van Hippel Lindau; p35 = tumor suppressor protein; hMSH2, MLH1 = DNA mismatch repair proteins; MYC = oncogene; PDGFRβ = platelet-derived growth factor receptor beta; wt = wildtype; mut = mutant; * low expression; ** high expression.

Table S3. Doses in clinical use for panobinostat (pan), vorinostat (vor), axitinib (axi), pictilisib (pic) and saracatinib (sar).

Drugs	CUD [μM]	Dose in ODC [μM]	Fold Change Between CUD and ODC
pan	10 [93]	10	–
vor	1.8 [94]	0.1	18
axi	0.2 [95]	0.02	10
pic	2 [96]	2	–
sar	0.6 [97]	0.6	–

CUD = clinically used dose; ODC = optimized drug combination.

Table S4. Combinatorial Index of three drug combination panobinostat (pan), vorinostat (vor), axitinib (axi) at various doses.

Pan [nM]	Vor [μM]	Axi [μM]	Effect	CI	Synergistic
10	1	0.2	0.838	6.08×10^{-3}	Yes
10	0.1	0.02	0.931	7.61×10^{-4}	Yes
10	0.5	0.02	0.923	9.99×10^{-4}	Yes
5	0.1	0.02	0.713	0.01	Yes
2.5	0.1	0.02	0.391	0.13	Yes

Effect = reduction of ATP levels (%CTRL); CI = Combination Index.

Table S5. Cross-validation of ODC in ccRCC and non-cancerous cell lines doses.

Cells	Caki-1	Caki-1-SR	786-O	786-O-SR	HEK-293T	NHDFα	ECRF24
Efficacy [%CTRL]	83.8	43.0	16.8	17.3	15.7	15.7	76.3
± SD	10.3	8.1	6.4	13.0	7.5	7.5	12.8

Table S6. 35 highest expressed transcripts of Caki-1 cells validated through RNA sequencing.

Transcripts	Full Name	Cell/Pathway Regulation	Raw count
CALR	Calreticulin	Protein translation	57953
CANX	Calnexin	Protein translation	56144
EEF1A1	Elongation factor 1-alpha 1	Protein translation	113929
EEF2	Elongation factor 2	Protein translation	61235
EIF4G1	Eukaryotic translation initiation factor 4 gamma 1	Protein translation	49814
EIF4G2	Eukaryotic translation initiation factor 4 gamma 2	Protein translation	70822
P4HB	Protein disulphide-isomerase	Protein post-translation	51965
PABPC1	Polyadenylate-binding protein 1	Protein translation	66324
RPL4	60S ribosomal protein L4	Protein translation	56108
RPLP0	60S ribosomal protein P0	Protein translation	53856
SLC7A5	Large neutral amino acids transporter small subunit 1	Protein translation	97678
YBX1	Y-box-binding protein 1	Protein transcription and translation	51395
ACTB	Actin, cytoplasmic 1	Attachment/Cytoskeleton	158937
ACTG1	Actin, cytoplasmic 2	Attachment/Cytoskeleton	216831
ACTR3	Actin-related protein 3	Attachment/Cytoskeleton	66785
FLNA	Filamin-A	Attachment/Cytoskeleton	112587
FN1	Fibronectin	Attachment/Cytoskeleton	83855
LAMC2	Laminin subunit gamma-2	Attachment/Cytoskeleton	41289
THBS1	Thrombospondin-1	Attachment/Cytoskeleton	
TUBA1B	Tubulin alpha-1B chain	Attachment/Cytoskeleton	
TUBB	Tubulin beta Class 1	Attachment/Cytoskeleton	
VIM	Vimentin	Attachment/Cytoskeleton	
ENO1	Alpha-enolase	Glycolysis/Homeostasis	86008
GAPDH	Glyceraldehyde-3-phosphate dehydrogenase	Glycolysis/Homeostasis	171569
PKM	Pyruvate kinase PKM	Glycolysis	127712
PKM	Pyruvate kinase PKM	Glycolysis	127712
FTH1	Ferritin heavy chain	Homeostasis	47432
LDHA	L-lactate dehydrogenase A chain	Homeostasis/pyruvate fermentation	93304
LDHB	L-lactate dehydrogenase B chain	Homeostasis/pyruvate fermentation	51734
HSP90AA1	Heat shock protein HSP 90-alpha Family Class A Member A1	Stress response	148152
HSP90AB1	Heat shock protein HSP 90-alpha Family Class B Member 1	Stress response	114277
HSPA8	(Heat Shock Protein Family A (Hsp70) Member 8	Stress response	85937

NCL	Nucleolin	Proliferation	54071
NPM1	Nucleophosmin	Proliferation	82341
HNRNPA2B1	Heterogeneous nuclear ribonucleoproteins A2/B1	RNA packaging	57384
AHNAK	Neuroblast differentiation-associated protein AHNAK		67386

Raw count 50,000 ≤ 240,000; gene names from UniProt.

Table S7. RNA expression of saracatinib (sar), pictilisib (pic), axitinib (axi) or crizotinib (cri) drug targets in Caki-1 and Caki-1-SR clone 1 cells.

Transcripts	Full Name	Cell/Pathway Regulation
MET		
MAP4K4	Mitogen-activated protein kinase kinase kinase kinase 4	Cell signaling (intracellular)
IRAK1	Interleukin-1 receptor associated kinase 1	Cell signaling (extracellular)
EPHA2	Ephrin type-A receptor 2	Cell signaling (extracellular)
GRB2	Grwoth factor receptor-bound protein 2	Cell signaling (intracellular)
EIF3J	Eucaryotic translation initiation factor 3 subunit J	Protein translation
PTK2	Focal adhesion kinase 1	Attachment/Cytoskeleton
YES1	Tyrosine-protein kinase Yes	Cell signaling (intracellular)
SLK	STE20-like serine/threonine-protein kinase	Stress response/Cell death
CSNK2A1	Casein kinase II subunit α	Cell signaling (intracellular)
CSNK2B	Casein kinase II subunit β	Cell signaling (intracellular)
ABL2	Tyrosine-protein kinase ABL 2	Cell signaling (intracellular)
UBASH3B	Ubiquitin-associated and SH3 domain-containing protein B	Protein degradation
AURKA	Aurora kinase A	Proliferation
INPPL1	Phosphatidylinositol 3,4,5-trisphosphate 5-phosphatase 2	Cell signaling (intracellular)
ABL1	Tyrosine-protein kinase ABL 1	Cell signaling (intracellular)
IRAK3	Interleukin-1 receptor associated kinase 3	Cell signaling (extracellular)
ACAD11	Acyl-CoA dehydrogenase family member 11	Cell signaling (intracellular)
LCK	Tyrosine-protein kinase Lck	Cell signaling (intracellular)
MST1R	Macrophage-stimulating protein receptor	Cell signaling (extracellular)
EPHA5	Ephrin type-A receptor 5	Cell signaling (extracellular)
FRK	Tyrosine-protein kinase FRK	Cell signaling (intracellular)
ACVR2B	Activin receptor type-2B	Cell signaling (extracellular)
PDGFRB	Platelet-derived growth factor receptor β	Cell signaling (extracellular)
TINK	NA	NA
TGFBR1	TGF-beta receptor type-1	Cell signaling (extracellular)
DDR1	Epithelial discoidin domain-containing receptor 1	Cell signaling (extracellular)

MAP3K1	Mitogen-activated protein kinase kinase kinase 1	Cell signaling (intracellular)
NQO2	Ribosyldihydronicotinamide dehydrogenase [quinone]	Stress response/Protein degradation
TNK2	Activated CDC42 kinase 1	Attachment/Proliferation
MAP4K2	Mitogen-activated protein kinase kinase kinase kinase 2	Cell signaling (intracellular)
FGFR1	Fibroblast growth factor receptor 1	Cell signaling (extracellular)
SRC	Proto-oncogene tyrosine-protein kinase Src	Cell signaling (intracellular)
AAK1	AP2-associated protein kinase 1	Endocytosis
BMPR1A	Bone morphogenetic protein receptor type-1A	Cell signaling (extracellular)
TANK	TRAF family member-associated NFκB activator	Cell signaling (intracellular)
SIK3	Serine/threonine-protein kinase SIK3	Cell signaling (intracellular)
FECH	Ferrochelatase (mitochondrial)	Cellular energy production
PRKD2	Serine/threonine-protein kinase D2	Cell signaling (intracellular)
EPHB4	Ephrin type-B receptor 4	Cell signaling (extracellular)
ADCK3	Atypical kinase COQ8A (mitochondrial)	Cellular energy production
ACVR1	Activin receptor type-1	Cell signaling (extracellular)
BMP2K	BMP-2-inducible protein kinase	NA
ACVR1B	Activin receptor type-1B	Cell signaling (extracellular)
SIK2	Serine/threonine-protein kinase SIK2	Cell signaling (intracellular)
RIPK2	Receptor-interacting serine/threonine-protein kinase 2	Cell signaling (intracellular)
EPHA4	Ephrin type-A receptor 4	Cell signaling (extracellular)
PLK4	Serine/threonine-protein kinase PLK4	Proliferation
MAP4K3	Mitogen-activated protein kinase kinase kinase kinase 3	Cell signaling (intracellular)
LINK2	Hyaluronan and proteoglycan link protein 2	Attachment/Cytoskeleton
IKBKE	Inhibitor of nuclear factor κB kinase subunit ε	Cell stress/inflammatory response
MAP4K5	Mitogen-activated protein kinase kinase kinase kinase 5	Cell signaling (intracellular)
BCR	Breakpoint cluster region protein	Cell signaling (intracellular)
EPHB2	Ephrin type-B receptor 2	Cell signaling (extracellular)
STK10	Serine/threonine-protein kinase 10	Cell signaling (intracellular)
AP2A1	AP-2 complex subunit alpha-1	Endocytosis
AURKB	Aurora kinase B	Proliferation
LIMK1	LIM domain kinase 1	Attachment/Cytoskeleton
LYN	Tyrosine-protein kinase Lyn	Cell signaling (intracellular)
GAK	Cyclin-G-associated kinase	Proliferation
CSNK2A2	Casein kinase II subunit α' (catalytical subunit)	Cell signaling (intracellular)
INCENP	Inner centromere protein	Proliferation

NA = not applicable; gene names from UniProt.

Table S8. HDACI combined with immune -modulatory regimens in clinical trials.

HDACI	IMA/ ICB	Cancer Type	Clinical Phase	ClinicalTrials.gov Identifier
Vorinostat	Pembrolizumab (anti-PD-1)	Advanced renal or urothelial cell carcinoma	Active Phase I/Ib	NCT02619253
Entinostat	Aldesleukin (IL-2)	Metastatic kidney cancer	Active Phase I/II	NCT01038778
CXD101	Nivolumab (anti-PD-1)	Metastatic colorectal cancer	Active Phase I/II	NCT03993626
Mocetinostat	Durvalumab (anti-PD-L1)	Advanced solid tumors and NSCLC	Completed Phase I/II	NCT02805660
Entinostat	Atezolizumab (anti-PD-L1)	Breast neoplasm	Recruiting Phase I/II	NCT03280563
Guadecitabine/Mocetinostat	Pembrolizumab	Advanced lung cancer	Recruiting Phase I	NCT03220477
Vorinostat	Pembrolizumab	Progressive advanced mucosal cancer of different locations	Recruiting Phase II	NCT04357873
Entinostat	Nivolumab/ Ipilimumab (CTLA-4)	Breast cancer	Active Phase I	NCT02453620

IMA = immune-modulatory agent; ICB = immune checkpoint blockade; PD-1 = programmed death-1 (surface protein of T cells); PD-L1 = programmed death ligand 1 (surface protein of cancer cells), NSCLC = non-small-cell lung carcinoma, CTLA4 = cytotoxic T-lymphocyte associated protein 4.

Table S9. Side effects of panobinostat (pan), vorinostat (vor), axitinib (axi) and pictilisib (pic) reported in the cardiovascular, central nervous, endocrine and gastrointestinal system and potential cross activities.

Drug	Reported Synergies with HDACI or TKI	Side Effects*	Side Effects in Combination with HDACI or TKI
pan	Erlotinib HCl° [17] Trametinib (MEK-inhibitor)°,°°° [98] Ponatinib° [99]	Abnormal T waves on ECG, Fatigue, Hypoglycemia, Diarrhea	Fatigue, Nausea, Rash, DLTs°°° [17]; Severe neuropathy°°° [100]
vor	Gefitinib°°,°°° [101,16]	Peripheral edema, Fatigue, Hyperglycemia, Diarrhea	Fatigue, No DLT°°° [102]; No considerable side effects°° [101]; Anemia, Fatigue, Diarrhea°°° [103,104]

axi	pic° [22,105]	Hypertension, Decreased serum bicarbonate, Abdominal pain, Fatigue	NA
pic	axi° [22,105]	Rash, Hyperglycaemia, Gastrointestinal symptoms, Fatigue [106]	NA

*Side effects were extracted from SIDER 4.1 Database (http://sideeffects.embl.de) and UpToDate database (https://www.uptodate.com/). MedDRA Preferred Term were preferred and only side effects with associated frequency at least equal to "very common" (or 10%) were kept.[107].Reported ° in vitro, °° in mice; °°° in patients; ECG = electrocardiography; DLT = dose-limiting toxicity; NA = not applicable

© 2020 by the authors. Licensee MDPI, Basel, Switzerland. This article is an open access article distributed under the terms and conditions of the Creative Commons Attribution (CC BY) license (http://creativecommons.org/licenses/by/4.0/).
